# Cationic Nanoparticle Networks (CNNs) with Remarkably Efficient, Simultaneous Adsorption of Microplastics and PFAS

**DOI:** 10.1021/acsami.4c21249

**Published:** 2025-02-10

**Authors:** Shayesteh Tafazoli, Dylan B. Shuster, Ali Shahrokhinia, Sahaj Rijal, Dorcas M. Ruhamya, Kamryn A. Dubray, David J. Morefield, James F. Reuther

**Affiliations:** † Department of Chemistry, University of Massachusetts Lowell, Lowell, Massachusetts 01854, United States; ‡ BASF Corporation, AMIC, DZ3, 1609 Biddle Avenue, Wyandotte, Michigan 48192, United States; § Department of Chemistry, Johns Hopkins University, Baltimore, Maryland 21218, United States

**Keywords:** microplastics, PFAS, polymerization-induced self-assembly, adsorption, cationic gels

## Abstract

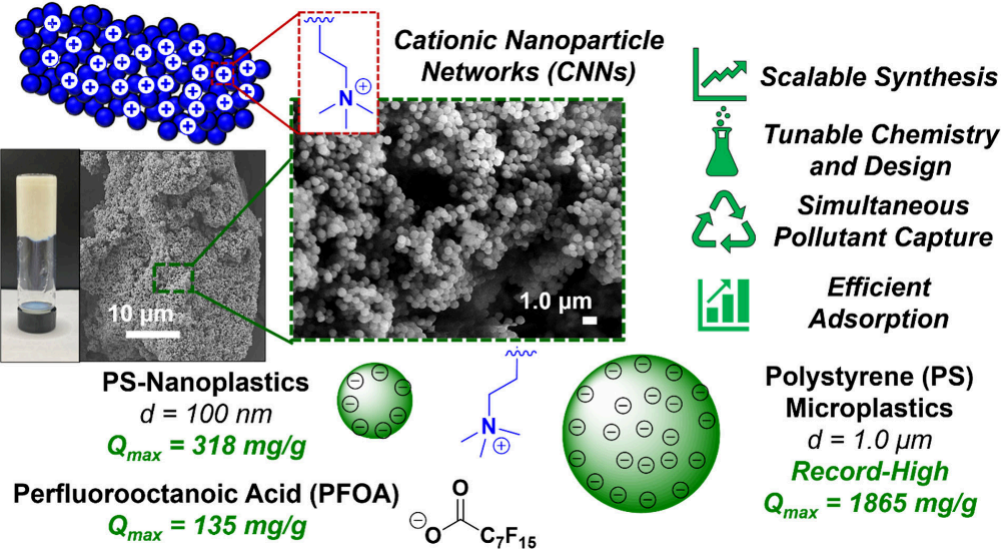

Of the past decade, micro/nanoplastics (MP/NP) and per- and polyfluoroalkyl substances (PFAS) have become two of the most pervasive persistent organic pollutants leading to significant accumulation within waterways. Various sorbent materials have been evaluated for PFAS and MP/NP removal, but their simultaneous removal has rarely been explored. Herein, we report a library of polymer-based, cationic nanoparticle networks (CNN) with systematic variation in surface charge density, polymer molecular weight, and nanoparticle size for the removal of anionic MP/NP and PFAS from aqueous solutions. These materials are synthesized in three, one-pot steps starting with polymerization-induced self-assembly (PISA) followed by rapid photocuring and quaternary ammonium salt formation resulting in 3D networks consisting solely of cationic polymer nanoparticles. Our best performing CNN material demonstrated record-high MP removal capacities of *Q*_max_ = 1865 mg/g and *K*_F_ = 58.0 (mg/g)(L/mg)^1/*n*^ based on Langmuir and Freundlich isotherm model estimations, respectively. Furthermore, the CNN materials demonstrated efficient removal of NPs and MPs in complex water media, such as in seawater and at different pH values, demonstrating the overall material applicability. Finally, simultaneous and efficient removal of MPs and perfluorooctanoic acid (PFOA) was accomplished with similar *Q*_max_ (MP) = 478.4 mg/g and *Q*_max_ (PFOA) = 134.6 mg/g allowing for dual use.

## Introduction

With less than 0.3% of Earth’s water considered “drinkable”, freshwater resources are limited.^1^ In 2022, the World Health Organization reported 2.2 billion people lacked consistent access to safely managed drinking water.^2^ Water security and contamination remain a critical global concern. The global consumption of plastic continues to grow (ca. 367 million tons since the middle of 20th century)^3^ with just ∼9% of the plastic waste recycled.^4^ Because of this, it is being predicted that ∼100–250 million tons of plastic litter will enter the water environment by 2025.^5^

Microplastics (MP; diameter (d) < 5 mm) and nanoplastics (NP; *d* < 1 μm) are generated through the chemical, microbiological, and/or mechanical degradation of larger plastic materials. These particles are pervasive in aquatic environments, presenting significant risks to both ecosystems and human health.^6^ It is estimated that individuals ingest, drink, and/or inhale millions of microplastic particles annually.^7^ Toxicological studies revealed how microplastics induce ROS generation, inflammation, immunosuppression, and neurological dysfunction, alongside their long-term risks such as infertility, immune disruption, and cancer.^8^

MP/NPs possess a high surface-to-volume ratio, which allow them to carry invasive microorganisms, heavy metals, and a range of toxic chemicals. This includes persistent organic pollutants such as pesticides, flame retardants, and per- and polyfluoroalkyl substances (PFAS).^9^ Many PFAS are environmentally persistent and bioaccumulative, having been found in human and animal tissues and bloodstreams worldwide, even in isolated populations. This accumulation is driven by both their widespread industrial and commercial use coupled with their exceptional thermodynamic stability. Exposure to PFAS can result in adverse health and environmental effects.^10^ Consequently, the potential health effects of MP/NPs and PFAS, along with their expected regulations, has led to increased need for transformative remediation technologies.^11,12^

For MPs, traditional drinking water treatment mainly includes coagulation, flocculation, sedimentation, sand filtration, and clarification. As a practical approach, different combinations of these methods have been applied to remove MPs with removal efficiencies between 66–89%.^12,13^ Advanced water treatment technologies such as electrocoagulation,^14^ magnetic extraction,^15^ and membrane separation^16^ have shown promise but many challenges hinder widespread implementation such as improvement of substrate scope, removal efficiency, fouling and operating costs.^12^ Furthermore, the presence of MPs in affected water streams can greatly affect downstream methods. For example, disinfection methods like chlorination, ozonation, and UV irradiation are hindered by microplastics, which can form biofilms and protect microorganisms, reducing overall treatment effectiveness.^17,18^ Thus, implementing advanced final-stage treatment technologies is essential to significantly mitigate microplastic pollution.^19^

Recent advancements in microplastic removal have introduced innovative strategies, including photoresponsive magnetic microrobots such as Ag@Bi_2_WO_6_/Fe_3_O_4_, capable of achieving 98% removal in 93 s,^20^ and self-propelled nanomaterials,^21^ like photophoretic MoS_2_–Fe_2_O_3_ micromotors which combine autonomous movement and pollutant degradation.^22^ However, these approaches generally rely on external energy inputs, such as magnetic fields, UV/visible light, or chemical fuels, for activation. Biochar-based materials, such as amino-functionalized zeolite/phosphoric acid-coffee waste biochar (AFZ), have been explored for the removal of polystyrene MPs. These materials are effective but have limited capacity compared to more advanced systems ranging from 4.78 to 4.85 mg/g.^23^

Metal–Organic Frameworks (MOFs), with nanoporous crystalline structures and modifiable surface properties, have demonstrated significant potential for microplastic removal.^24^ Cr-MOF/MIL-101, for example, has shown a 96% removal efficiency for polystyrene nanoplastics (70 ppm) through electrostatic and π–π interactions. Despite their large surface area, MOFs often face challenges related to pH sensitivity.^25^ The efficiency of MOFs like Zr-UiO-66^26^ relaying on the positive charges at specific pH ranges (such as pH < 5.5), decreases under more alkaline conditions, which are prevalent in seawater. While MOFs possess high surface areas, their practical application is hindered by the limited availability of accessible metal sites in some structures, reducing the interaction between metal ions and target microplastics. Moreover, synthesis processes are complex, and the formation of ideal structural frameworks is crucial for optimizing performance.^27^

Biological materials, such as amyloid fibrils,^28^ and membrane bioreactors offer promising strategies for microplastic removal. However, protein-based fibrils are sensitive to environmental factors like pH, temperature and salinity,^29^ while membrane bioreactors, despite achieving complete pollutant removal, face challenges such as fouling, aeration requirements, and high costs.^30^ Similarly, fungal bioremediation demonstrates potential through microplastic-trapping pellets^31^ but is limited by competition with native bacteria, environmental unpredictability, and scalability concerns.

For PFAS remediation, granular activated carbon (GAC), ion exchange resins (IX), and reverse osmosis (RO) have been recognized as potential solutions by the EPA and other environmental agencies.^32^ None of these technologies, however, are considered comprehensive solutions for achieving high-performance removal of PFAS in all cases. For instance, while RO can remove up to 99% of many PFAS,^33^ these methods are not selective separation processes, are prone to fouling, and require significant energy input.^34^ Traditionally, different filters which target specific pollutants are connected to enable stepwise water purification. Filter materials typically include porous adsorbents, such as zeolites, minerals, or granular sorbents such as as GAC and IX.^35,36^ All of these materials and methods are limited, however, by low selectivity, limited regenerability, and slow sorption kinetics.^37^ However, treating large volumes of water requires alternative methods that combine ease of use, minimal technological requirements, and the ability to simultaneously remove multiple contaminants.

Recent developments in the removal of PFAS have led to a variety of innovative materials. For example, He et al. reported a fluorinated nonporous adaptive crystalline cage (F-Cage 2), utilizing electrostatic interactions, hydrogen bonding, and F–F interactions, with a maximum PFOA adsorption capacity of 45 mg/g.^38^ Additionally, a zirconium-based MOF (PCN-999) demonstrated an exceptional PFOA uptake of 1089 mg/g (2.63 mmol/g), a nearly 50% increase over the previous MOF record, although its performance is limited to just PFOA removal.^39^ Covalent organic frameworks (COFs), particularly imine-linked 2D COFs with primary amines, have also shown great promise in removing contaminants like GenX, exhibiting rapid adsorption at environmentally relevant concentrations.^40^ Furthermore, functionalizing thin-film composite (TFC) hollow fiber nanofiltration (HFN) membranes with MXene nanosheets has improved the removal of perfluorooctanesulfonic acid (PFOS), primarily through electrostatic interaction and size exclusion mechanisms.^41^ All-silica zeolite β has also been identified as an alternative adsorbent with high selectivity and capacity for both PFOA and PFOS removal.^42^ Other promising adsorbents include cross-linked β-cyclodextrin (β-CD)^43^ polymers and quaternary ammonium cation-doped carbon nanoparticles (QACNs) for efficient PFAS removal from contaminated water sources.^44^ However, most of these materials are either limited to removing only PFAS or exhibit restricted capacity for widespread applications.

To the best of our knowledge, no technique has demonstrated the ability to remove both MP/NPs and PFAS with high efficiency, high capacity, and fast kinetics, simultaneously. Herein, we detail the synthesis and implementation of cross-linked cationic nanoparticle networks (CNNs) comprised of polymer nanoparticles covalently cross-linked into 3D nanostructured thermosets. This synthesis starts with photocontrolled atom transfer radical polymerization-induced self-assembly (PhotoATR-PISA), which generates polymer nanoparticles at high concentrations. This established technique is widely used for synthesizing nanoparticles with our previous studies focused on optimized parameters, morphology, and monomers for various applications.^45−48^ Following the synthesis of the nanoparticles, rapid photocuring can be achieved in a one-pot process. Methylation of tertiary amine pendants into quaternary ammonium salts yield CNNs with high removal efficiencies and remarkable adsorption capacities for MP/NPs from various aqueous environments (e.g., pure water, seawater, caustic water). Furthermore, simultaneous adsorption/flocculation of perfluorooctanoic acid (PFOA) and MPs was demonstrated providing unique opportunities for dual use applications in water treatment industries.

## Materials and Methods

Cupric bromide (Cu(II)Br_2_, 99%, Sigma-Aldrich), propargyl alcohol (Sigma-Aldrich), triethylamine (Fisher Scientific), 2-bromoisobutyryl bromide (Sigma-Aldrich), methacryloyl chloride (Fisher Scientific), and tris(pyridin-2-ylmethyl) amine (TPMA, TCI America), tris[2-(dimethylamino)ethyl]amine (Me_6_TREN, Sigma-Aldrich), and methyl iodide (CH_3_I, TCI America), Perfluorooctanoic acid bromide (Sigma-Aldrich), were used as received. All solvents have been purchased from Fisher Scientific and were used as received. HPLC-grade THF was purchased (Fisher Scientific) and used as received for GPC analysis. 2,2,2-Trifluoroethanol (TFE, Sigma-Aldrich) was used for macroinitiator synthesis. Prior to use, 2-(dimethylamino) ethyl methacrylate (DAEMA, Sigma-Aldrich), oligo(ethylene glycol) methyl ether methacrylate (OEGMA, *M*_n_ = 300 Da, Sigma-Aldrich), benzyl methacrylate (BMA; Alfa Aesar), and 1,6-hexanediol dimethacrylate (HDMA, TCI America) were passed through a column of basic alumina to remove radical inhibitors.

Yellow-green fluorophore-labeled polystyrene microparticles (PS-MP, diameter = 1.0 μm) with carboxylic acid functionality, used as fluorescent microplastic mimics, were procured from Invitrogen by Thermo Fisher. These PS particles possessed excitation and emission wavelengths of 505 and 515 nm, respectively. To disperse the PS spheres in water, a sonicator bath was employed. Additionally, nonfluorescent polystyrene nanoparticles (PS-NPs, diameter = 100 nm), serving as a model for nanoplastics, were obtained from Lab 201 California. PS-NPs also featured carboxylic acid functionality on their surface to mimic the surface charge of naturally formed NPs. Synthesis of alkyne-ATRP initiator was adapted from previously reported literature.^45^ A XICHEN 36W UV nail gel curing lamp (available from a variety of suppliers such as WalMart or eBay) (max 360 nm) with four 9W bulbs used for all polymerizations.

### Synthesis of PDAEMA(D)-Based Macroinitiator

Two distinct macroinitiators with different degrees of polymerization (DP) were successfully synthesized using the same procedure. As an example, purified 2-(dimethylamino)ethyl methacrylate (DAEMA) monomer (5.0 mL, 29.6 mmol, 80 equiv), alkyne-based ATRP initiator (75.9 mg, 0.370 mmol, 1.0 equiv), CuBr2 (1.6 mg, 7.4 μmol, 0.02 equiv), Me6TREN (10.23 mg, 44.4 μmol, 0.12 equiv), and 5.0 mL of solvent, 2,2,2-trifluoroethanol (TFE) were added to a septum sealed vial and degassed with nitrogen for 15 min. Polymerization started upon placing the degassed reaction mixture under UV irradiation and was quenched at ∼60–70% PDAEMA conversion to preserve end-group fidelity by exposure to air. After removing TFE by rotary evaporation, the macroinitiator was diluted with a small amount of DCM (∼2 mL) and passed through a column of neutral alumina to remove residual copper catalyst. Then, excess DCM was removed by rotary evaporation and the viscous macroinitiator was precipitated from cold hexanes. The chemical structure and molecular weight of the macroinitiator were confirmed using ^1^H NMR spectroscopy and GPC (Figure S1).

### Synthesis of POEGMA-Based Macroinitiator

A similar methodology as described above was employed, instead substituting purified OEGMA, to synthesize a control NN gel without cationic character. First, purified OEGMA monomer (5.0 mL, 17.5 mmol, 40 equiv), alkyne-based ATRP initiator (89.7 mg, 0.4375 mmol, 1.0 equiv), CuBr2 (1.95 mg, 8.75 μmol, 0.02 equiv), Me6TREN (12.1 mg, 52.5 μmol, 0.12 equiv), DMF (internal standard; 325 μL, 4.2 μmol), and 5.0 mL of TFE solvent were added to a septum sealed vial and degassed with nitrogen for 15 min. Polymerization started upon placing the degassed reaction mixture under the UV irradiation and was quenched at ∼60–70% OEGMA conversion to preserve end-group fidelity. After removing TFE by rotary evaporation, the macroinitiator was diluted a small amount of DCM (∼2 mL) and passed through a column of neutral alumina to remove residual copper catalyst. Then, excess DCM was removed by rotary evaporation and the viscous macroinitiator was precipitated from cold diethyl ether: hexanes mixture (50:50 by volume). The chemical structure and molecular weight of the macroinitiator were confirmed using 1H NMR spectroscopy and GPC (Figure S2).

### Synthesis of Cationic Nanoparticle Networks (CNN) Using Two-Pot PhotoATR-PISA and Photocuring

Five distinct gels were synthesized utilizing three different macroinitiators (Table S1). Among these, one gel served as a control and was formulated using P macroinitiators (NN5), while the remaining gels were synthesized using D macroinitiators with tertiary amine pendants (NN1–4). The synthesis of all NN gels was carried out using the same procedure with the following protocol. For synthesis of NN4 below (Scheme S1), the desired amount of macroinitiator (Mn = 12.3 kDa, 300 mg, 24.4 μmol, 1.0 equiv), BMA (1.239 mL, 7.3 mmol, 300 equiv), CuBr2 (0.05 mg, 0.244 μmol, 0.01eq), TPMA (0.283 mg, 0.976 μmol, 0.04 equiv), and methanol for SC% = 25 w% (8.02 mL) were added to the septum sealed vial and degassed by purging with nitrogen for 15 min. The polymerization was then started upon placing the degassed reaction mixture under UV irradiation monitoring monomer conversion via 1H NMR until ∼90% PBMA conversion. At this time, 1,6-hexanediol dimethacrylate (HDMA) was degassed with N2 for 15 min and introduced to the reaction mixture as a cross-linker (0.558 g, 2.19 mmol, 30 mol % with respect to BMA). The reaction proceeded under UV irradiation until full monomer conversion for PhotoATR-PISA and was terminated upon exposure to air. Gels were worked up upon washing with methanol and centrifuging at 7,000 rpm for 5 min, a procedure that was repeated four times to remove unreacted monomer and cross-linker. The gels were then dried in a vacuum oven overnight at 40 °C. Samples were taken periodically and analyzed using ^1^H NMR and GPC for kinetic analysis (Table S1 and Figure S3). Transmission electron microscopy (TEM) was employed to analyze the morphology evolution of the PISA1–5 nanoparticles prior to cross-linking, while scanning electron microscopy (SEM) was utilized to examine the surface morphology of the purified vacuum-dried NN1–5 gels

### Methylation of PDAEMA (D) Tertiary Amine Pendants for CNN Ion-Exchange Adsorbents

To facilitate the process of amine methylation postpolymerization, the gel was dispersed in a 10 mL TFE solution within a 100 mL round-bottom flask. Subsequently, the desired quantity of CH_3_I (100 equiv relative to the amine group content, as determined by ^1^H NMR analysis) was introduced into the flask, and the reaction was allowed to proceed for 72 h. After the completion of the reaction, the surplus trifluoroethanol (TFE) was removed through centrifugation and decanting, Methanol was employed as a solvent for the purpose of conducting washing procedures employing methanol as a medium. Subsequently, the resultant mixture was subjected to vacuum oven drying overnight.

## Results and Discussion

### Design and Structural Characterization of Cationic Nanoparticle Networks (CNNs)

**Figure 1:**
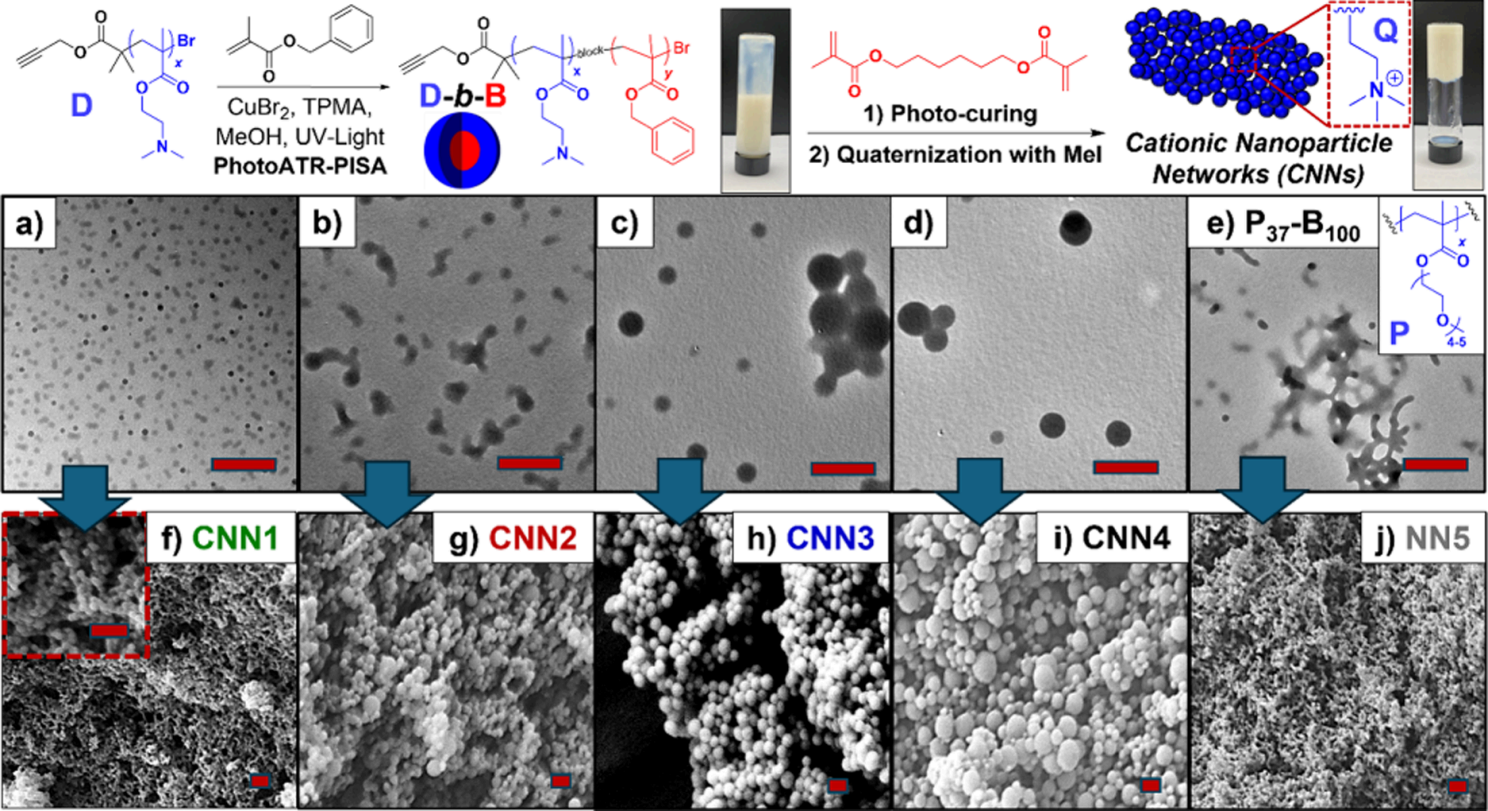
One-pot synthesis of CNNs starting with PhotoATR-PISA of D-*b*-B block copolymer nanoparticles followed by bismethacrylate photocuring and methylation with methyl iodide. Morphologies of PhotoATR-PISA nanoparticles were characterized via TEM showing increases in spherical micelle diameter as molecular weights increase in PISA1(D_35_B_100_) (a), PISA2(D_75_B_100_) (b), PISA3(D_35_B_300_) (c), and PISA4(D_75_B_300_) (d). Upon photocuring with HDMA and methylation/quaternization of D pendant groups (Q), cationic nanoparticle networks (CNNs) with varying 3D network structures were characterized via SEM including CNN1(Q_35_B_100_) (f), CNN2(Q_75_B_100_) (g), CNN3 (Q_35_B_300_) (h), and CNN4(Q_75_B_300_) (i). One control, nonionic nanoparticle networks (NN) was also synthesized starting with PhotoATR-PISA from P macroinitiators (PISA5(P_37_B_100_)) (e) and photocuring forming NN5(P_37_B_100_) (j). All scale bars = 500 nm.

The synthesis of the reported CNN materials occurred in 4 steps starting with macroinitiator synthesis and workup, followed by PhotoATR-PISA, photocuring and tertiary amine methylation in one-pot (Figure 1). Initially, poly(dimethylaminoethyl methacrylate) (D)-based macroinitiators were synthesized at two different, targeted degrees of polymerization (DP(D) = 40 and DP(D) = 80). To maintain end-group fidelity, these polymerizations were quenched at approximately 60–70% D conversion. These macroinitiators were characterized using GPC and ^1^H NMR analysis (ca. *M*_n_ = 6.62 kDa, *Đ* = 1.12, DP = 35 and *M*_n_ = 12.3 kDa, *Đ* = 1.12, DP = 75; Figure S1). A control stabilizer sample was synthesized by replacing the basic tertiary amine pendants of D stabilizers with poly(oligoethylene glycol methacrylate) (P) block with *M*_n_ = 5.10 kDa, *Đ* = 1.45, DP = 38 (Figure S2).

Subsequently, poly(benzyl methacrylate) (B) was utilized as a core-forming block for PhotoATR-PISA with targeted DP(B) = 100 and 300 at solids content = 25 w% using both macroinitiators. The polymerization process was continued until approximately 80–90% monomer conversion based on ^1^H NMR analysis (Figure S3). GPC and NMR analysis were performed at this stage prior to cross-linking to calculate MW but consumption of remaining B monomer is expected during cross-linking. A clear shift toward higher molecular weight (MW) indicated the growth of polymer chains and formation of the desired block copolymer (Scheme S1 and Table S1). As the MW increases in the four D-*b*-B PhotoATR-PISA nanoparticles, we observed spherical micelle morphologies of increasing size rather than morphological evolution, as evidenced by TEM (Figures 1a–1d). Furthermore, a control PISA nanoparticle sample was synthesized with poly(oligoethylene glycol methacrylate) block (P; Figure 1e). PhotoATR-PISA with P macroinitiators and B core-forming blocks resulted in the formation of small spherical micelles and nanoworms.

Upon achieving approximately 80–90% B conversion in all cases, the cross-linker, 1,6-hexanediol dimethacrylate (HDMA), was introduced at target DP(HDMA) = 30 mol % of core-forming monomers, resulting in the transition of the polymer nanoparticle dispersions into an arrested gel state. For this, propagation of cross-linker begins in the core of the nanoparticles and extends outward to the periphery of the nanoparticles allowing for both inter- and core-nanoparticle cross-linking, simultaneously. This gelation mechanism led to the preservation of morphology for polymeric nanoparticles, as evidenced by SEM (Figures 1f–1j). These interconnected 3D networks were hypothesized to possess a porous structure attributed to the presence of interstitial spacing between nanoparticles which could facilitate small molecule adsorption and removal through tortuosity. Following gelation, methyl iodide was employed to methylate the tertiary amine pendants of D blocks, thereby introducing cationic ammonium iodide functionalities (Q) forming CNN ion exchange adsorbents.

The morphology of all PISA nanoparticles and CNN gel adsorbents were characterized using TEM and SEM, respectively. Prior to the introduction of the HDMA cross-linker, in all cases, Dx-By PISA nanoparticles displayed spherical micelle morphologies. As the MW increases, we observed an increase in nanosphere diameters with d = 46.6 ± 7 nm, 149 ± 45.6 nm, 187 ± 19 nm, and 239 ± 73 nm transitioning from PISA1(D_35_B_100_) to PISA4(D_75_B_300_) (Table S2). These size increases correlate well to nanoparticles in CNN materials following cross-linking and methylation, except with some diameter growth during cross-linking, with d = 84 ± 9.7 nm, 206 ± 36.9 nm, 279.39 ± 50.4 nm, and 315.45 ± 70.7 nm transitioning from CNN1(Q_35_B_100_) to CNN4(Q_75_B_300_). For control PISA5(P_37_B_100_) and NN5(P_37_B_100_), the small nanoworm morphologies were measured with lengths = 106.3 ± 49.8 nm and d = 115.5 ± 25.3 nm as measured by TEM and SEM, respectively (see Figure S4 for zoomed out images). BET surface area analysis of CNN1–4 and NN5 materials demonstrates variable porosity with surface area *S*_BET_ = 12.2–42.6 m^2^/ g (Figure S5) with CNN4(Q_75_B_300_) showing the highest surface area of all samples via N_2_ adsorption isotherms (Table S3). The average pore diameter (D_BET_) was also calculated via BET measurements with D_BET_ = 53.6, 32.3, 15.5, 45.0 nm for CNN1–4, respectively and the pore size distributions for CNN1–4 are presented in (Figures S6–S10).

### MP and NP Removal Efficiency and Adsorption Kinetics

**Figure 2:**
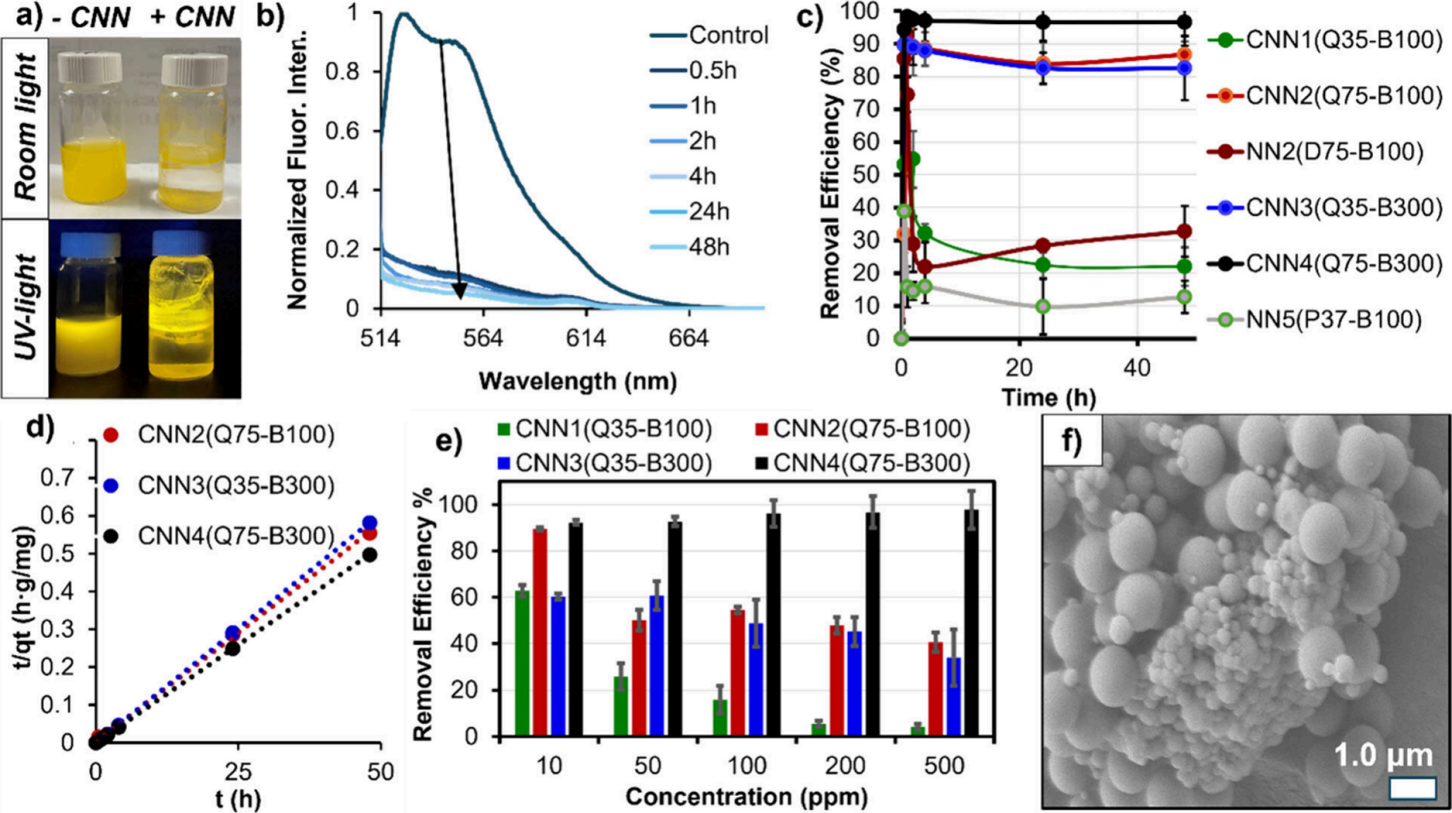
Photo of fluorescent PS-MP dispersion with (right) and without (left) CNN gels under room light (top) or UV-light (bottom) irradiation (a). Fluorescence spectroscopy (b) was used to quantitatively monitor the removal of PS-MPs over the course of 48 h for CNN1–4, NN2, and NN5 gel adsorbents (c; [PS-MP] = 2.00 ppm). Using this, the adsorption kinetics were fit to pseudo-second-order kinetic models with CNN-4 demonstrating the best adsorption rates (d). RE was measured for all CNN samples from [PS-MP] = 10–500 ppm for isotherm calculations (e), and SEM analysis of the CNN4 gels after adsorption ([PS-MP] = 150.0 ppm) showed the presence of MPs bound to the surface of gels (f).

To measure and monitor the removal efficiencies (RE) of MPs and NPs using CNN materials, either fluorescent polystyrene microspheres (PS-MP; d = 1.0 μm) or nonfluorescent PS nanospheres (PS-NP; d = 100 nm) were used as representative models. The sizes and negative surface charge (i.e., from carboxylate surface functions) of these mimics correlate closely to typical dimensions and functionality of naturally formed MP/NPs.^49,50^ Both mimics displayed negative Zeta-potential as measured by DLS (ca. −100.0 mV and −95.8 mV) for MPs and NPs, respectively (Figure S11 and Table S4). This negative surface charge mirrors MP/NP contaminants found in the environment which often feature anionic character due to oxidative surface reactions.^51^ The fluorescent nature of the PS-MP mimics allows for naked-eye observation of MP removal (Figure 2a) and quantification of Removal Efficiency (RE) using fluorescence spectroscopy (Figure 2b).

To assess the purification potential of quaternary ammonium-based CNN1–4 gels compared to a control, nonionic NN5 gel with P shell-forming blocks, 10.0 mg of each gel was implemented for batch adsorption experiments. Initial experiments measured RE of 2.00 ppm PS microplastics for all adsorbents across a 48-h window (Figure 2c). The removal of MPs using nonionic NN5(P_37_B_100_) gel was found to be very low with RE = 12.6% after 48 h. In contrast, all but one positively charged CNN gels exhibited removal efficiencies that are at least three times higher. This stark difference highlights the critical importance of electrostatic interactions for enhancing adsorption of PS-MPs with negative surface charges. This is further exemplified by the lower overall adsorption of PS-MPs for NN2(D_75_B_100_) using the same CNN structure prior to methylation of D pendant groups. For these materials, we observe an initial spike in RE = 85.4% after 30 min but this quickly equilibrates to an RE = 32.7% after 48 h which can be attributed to the reversible nature of the binding. CNN1(Q_35_B_100_), with a short Q shell-blocks and low MW B core blocks, reached RE = 21.9% after 48 h with relatively slow adsorption kinetics. In comparison, CNN2(Q_75_B_100_), which features a higher MW cationic shell, demonstrated a maximum RE = 95.6% after 30 min and equilibrium RE = 86.7% after 48 h. The removal efficiencies observed for CNN3(Q_35_B_300_) demonstrated a similar trend as CNN2 reaching a maximum RE = 89.6% after 30 min and equilibrium RE = 82.6% after 48 h. CNN4(Q_75_B_300_) gel exhibited near quantitative RE = 98.2% for PS-MP after 30 min which remained largely stable over the course of 48 h with RE = 96.6%.

Based on these results, the MP adsorption kinetics were analyzed and modeled (Figure 2d). This removal was fit to pseudo-second-order kinetic models (Equation S3) leading to calculations of kinetic parameters (Table S5). We observed that CNN4(Q_75_B_300_) exhibited the highest adsorption rate constant with *k*_2_ = 1.35 g(mg^–1^)(min^–1^) indicating that adsorption equilibrium is rapidly reached. These values are ∼10 times the next highest values demonstrating the clear advantages of CNN4. The slowest kinetics were observed for noncharged NN5(P_37_B_100_) providing further evidence of weaker interactions between the adsorbate and the adsorbent,^52^ which may imply that the process is more diffusion-limited.^53^ The RE for PS-MPs were then analyzed for CNN1–4 across a wide range of concentrations (ca. [PS-MP] = 2–500 ppm) to determine maximum capacities for each adsorbent when modeled with typical adsorption isotherms (vide infra; Figure 2e). Remarkably, CNN4(Q_75_B_300_) demonstrated near quantitative RE (ca. 93–97%) across all concentrations analyzed. SEM analysis of CNN4 following exposure to 150 ppm PS-MP solutions show many surface-bound MPs confirming that electrostatic-driven surface interactions drive the adsorptive removal of the PS-MPs (Figure 2f).

**Figure 3:**
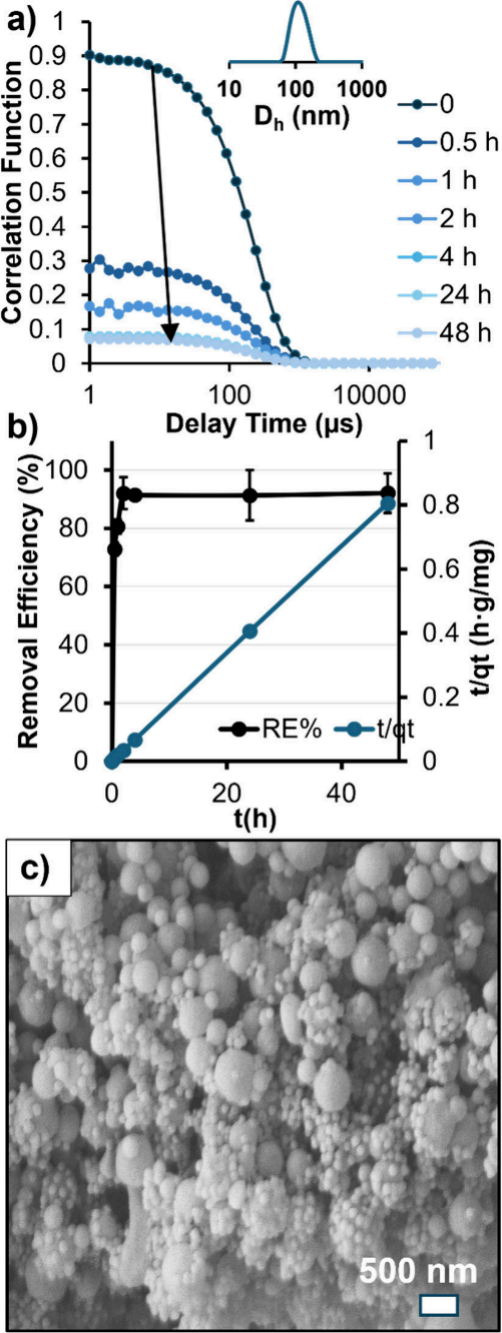
Removal of PS-NP (c = 2.00 ppm) using CNN4(Q_75_B_300_) was monitored and quantified using DLS-derived count rates and decreases in correlation function (a) to provide RE over a 48-h window (b). Pseudo-second-order kinetics were used to model parameters and SEM provided evidence for PS-NP surface binding similar to PS-MPs (c).

To investigate the removal of PS-NP mimics (d = 100 nm), DLS was used to quantify RE. DLS measurements, reported as “DLS derived count rate” in kilo-counts per second (kcps) were utilized to determine nanoplastic concentration based on photons scattered and are normalized to 0% attenuation at 100% laser power.^54,43^ Uniform measurement parameters were used for consistency. This removal can also be visualized and quantified using the correlation function plots from DLS showing diminishing intensities as PS-NPs are removed (Figure 3a). The top-performing gel, CNN4(Q_75_B_300_), was employed in initial batch adsorption experiments with 2.00 ppm PS-NP dispersions (Figure 3b). After 48 h, the gel achieved RE = 92.1%, reaching equilibrium approximately after ∼4 h. This corresponds to kinetic parameters, again obtained via modeling pseudo-second-order kinetics, with *k*_2_ = 0.217 g(mg^–1^)(min^–1^). SEM of CNN4 gels after adsorption of 150 ppm PS-NP confirmed the presence of small PS-NPs on the gel surface, similar to PS-MPs but this time with the smaller particles as the adsorbed contaminant (Figure 3c).

To demonstrate real-world applicability, it is important to assess aqueous matrix effects on overall removal efficiencies. For this, we first investigated the removal of 50 ppm PS-MP using CNN4(Q_75_B3_00_) at different pH = 3, 7, 8, and 9 as well as seawater (Figure S14). The optimal pH for removal was found to be pH = 8 with RE = 90.3%. Furthermore, the removal of PS-MPs (c = 50 ppm) was also investigated in simulated seawater with high salinity. This experiment is particularly important due to the large amount of plastic pollution that exists in Earth’s oceans.^55^

For this, we measured a RE = 89.6% when removing 50 ppm PS-MPs demonstrating the versatile application of CNNs in various aqueous environments. We also examined the effects of washing saturated CNN gels with various salt solutions to examine the stability of the CNN-MP complexes. Two different salts (i.e., NaCl and ammonium acetate) were investigated to break the electrostatic complexes through charge screening. This proved largely ineffective at concentrations <1 M with overall ∼18.1% and 34.3% of PS-MP desorbed upon washing with 1 and 6 M NaCl solutions, respectively. The strength of the interactions, even in high salinity aqueous environments, exemplifies the performance of CNN4 for the removal of PS-MP contaminants (Figure S15).

### Adsorption Isotherms for PS-MP and PS-NP Removal

To better understand the adsorption behavior of CNN gels, we utilized the Langmuir and Freundlich isotherm models. The adsorption characteristics of each gel were evaluated by monitoring their removal efficiency at varying pollutant concentrations (Figure 2e). The Langmuir and Freundlich isotherm models are widely used to describe the sorption behavior of solutes onto solid surfaces. These models provide mathematical equations that relate the equilibrium concentration of the solute in the solution to the amount of solute adsorbed onto the sorbent material. The Langmuir isotherm model (eq S4) assumes monolayer adsorption onto a homogeneous surface, where adsorption occurs at specific sites on the sorbent material. It proposes that the maximum adsorption capacity (*Q*_max_) is reached when all available adsorption sites are occupied, and the K_L_ value is related to the affinity of the solute for the sorbent surface. The Freundlich isotherm model (eq S5) is an empirical equation that accounts for multilayer adsorption and assumes nonuniform distribution of adsorption sites on the sorbent surface. *K*_F_ is the Freundlich constant related to adsorption capacity, and n is the Freundlich exponent that indicates the intensity of adsorption. A value of *n* > 1 suggests favorable adsorption, while a value <1 indicates weaker adsorption. Both the Langmuir and Freundlich models have their own assumptions and limitations and are typically applied adsorption of soluble constituents.^56^ These models, however, were still implemented for dispersed MP/NP mimics to estimate the total capacities associated with their removals due to the similar chemisorptive surface interactions that drive removal.

**Figure 4:**
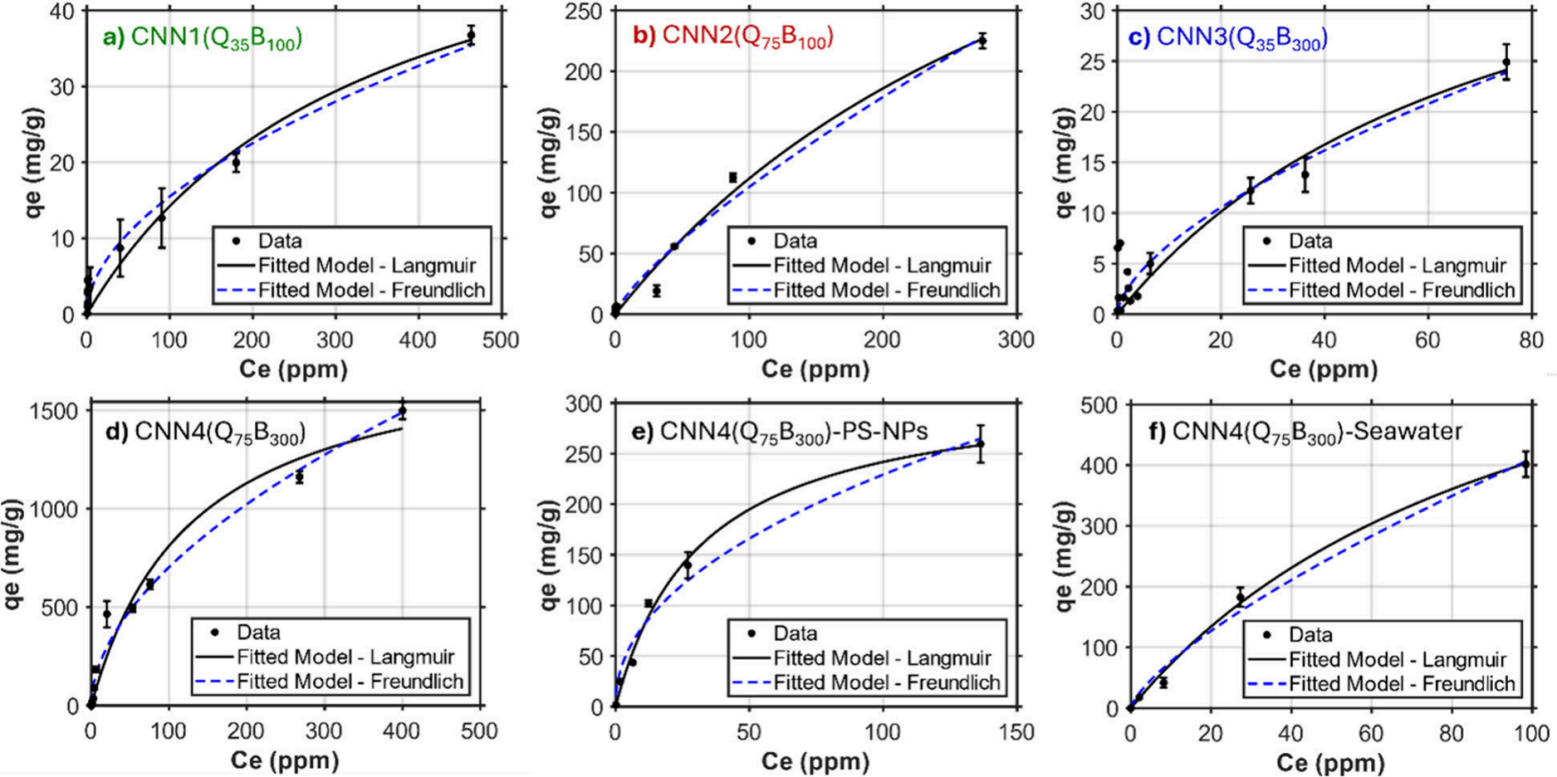
Adsorption isotherms for PS-MP mimics using CNN1–4 (a–d) fit to Langmuir and Freundlich isotherm models for quantification of adsorptive capacities for all gels. This analysis was also performed for removal of PS-NPs from tap water (e) and PS-MPs from simulated seawater (f) using our top performing CNN4(Q_75_B_300_) adsorbent material.

IntroductionAll calculated values related to each model are summarized in Table S6. Each isotherm model was applied to CNN1–4 using the batch adsorption experiments previously discussed (Figure 4). As expected, CNN4(Q_75_B_300_) exhibited the highest adsorption capacity with a remarkable *Q*_max_ = 1865 mg/g obtained from Langmuir isotherm fits, and *K*_F_ = 58.1 (mg/g)(L/mg)^1/*n*^ and *n* = 1.85 obtained from Freundlich isotherm models. The *Q*_max_ value measured, to the best of our knowledge, represented the highest known value for MP removal capacities when compared to other examples in literature.^57−60^ The R2 values for both models are similar (>0.97), indicating that either model may provide relatively accurate estimations. These values are also supported by the high RE values obtained even when expanding our experiments to high concentrations (*c* > 200 ppm) and lower adsorbent loadings (c = 500 ppm with 2.0 mg of CNN4). In comparison, CNN2(Q_75_B_100_) provided the next highest *Q*_max_ = 533 mg/g, *K*_F_ = 3.29, and *n* = 1.32, with the Langmuir model showing a higher R2 = 0.990. This further exemplifies the driving forces behind removal are facilitated by larger amounts of CNN positive surface charge due to the longer stabilizer blocks. CNN1 and CNN3 demonstrated relatively low adsorption capacity values with *Q*_max_ = 62.7 and 48.5 mg/g, respectively.

To examine the adsorptive performance of CNN4 in other aqueous environments, we conducted similar PS-MP isotherm calculations in simulated seawater with high salinity due to the high quantity of plastic pollution in the oceans. This analysis provided slightly diminished but still impressive adsorption capacities with *Q*_max_ = 822 mg/g and *K*_F_ = 14.4 (mg/g)(L/mg)^1/*n*^, respectively, demonstrating applicability in diverse aquatic environments (Figure 4f). Due to the high performance, we also selected CNN4 for PS-NP removal analysis and isotherm calculation. For this, CNN4 again demonstrated impressive adsorptive capacities for PS-NPs, though lower than PS-MPs, with *Q*_max_ = 318 mg/g and *K*_F_ = 26.9 (mg/g)(L/mg)^1/*n*^. Comparatively, these values are competitive to other materials reported in literature. For example, granular activated carbon (GAC) exhibits a maximum adsorption capacity of *Q*_max_ = 2.20 mg/g for the removal of PS-NPs, primarily through electrostatic interactions.^61^ Additionally, biochar derived from sugar cane bagasse demonstrates optimal PS-NP adsorption capacities of *Q*_max_ = 44.9 mg/g when prepared at 750 °C.^62^ Fly ash, modified with iron ions, produces novel magnetic materials (NMA) effective in removing PS-NPs from aqueous solutions, achieving adsorption capacities between *Q*_max_ = 82.8 and 89.9 mg/g at pH = 5–7.^63^ Composite adsorbents composed of MgAl-layered double oxides (LDO) integrated with biochar were the only found example with higher PS-NP adsorption capacity reaching up to *Q*_max_ = 360 mg/g at temperatures ranging from 30 to 50 °C.^64^

### Simultaneous Removal of PS-MPs and PFOA

MPs and PFAS, due to their global distribution, persistence, strong bioaccumulation and potential toxicity, are pervasive environmental pollutants resulting from both consumer and industrial activities.^65^ Additionally, MPs are known to transport other contaminants, including PFOA, which may affect the transport dynamics of both pollutants.^66^ The public’s increasing apprehension surrounding environmental and health impacts of these pollutants, coupled with the lack of research on the simultaneous removal of MPs and PFOA from water, underscores the relevance of this study.

**Figure 5:**
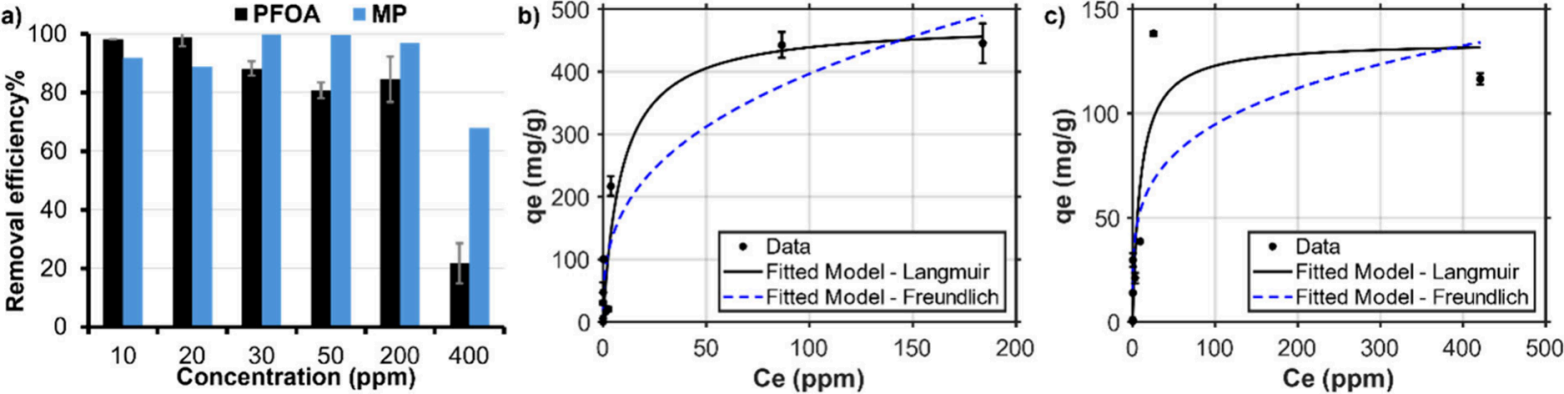
Simultaneous adsorption of PFOA and PS-MPs using CNN4(Q_75_B_300_) over a range of starting concentrations ([PFOA] = [PS-MP] = 10–400 ppm) (a) which were used to calculate adsorption isotherms for PS-MP (b) and PFOA (c) fit to both Langmuir and Freundlich models. These models were used to measure overall adsorption capacity during simultaneous removal.

The application of CNNs with tunable porous structures offers a promising avenue for the removal of small molecules and suspended impurities from water. Due to the impressive performance with isolated contaminants, CNN4(Q_75_B_300_) was chosen for simultaneous removal experiments keeping both concentrations constant in each experiment (i.e., [PFOA] = [PS-MP]). This material demonstrated high removal efficiencies at elevated concentrations, achieving RE(PS-MP) = 91.8%, 99.8%, 99.7% 96.9%, and 67.8% at [PS-MP] = 10, 30, 50, 200, and 400 ppm, respectively (Figure 5a). Isotherm analysis reveals a significant adsorption capacity for microplastics (MP) in the mixture of MP and PFOA, with the Langmuir and Freundlich model predicting a maximum adsorption capacity of *Q*_max_ = 478.4 mg/g and *K*_F_ = 80.45 (mg/g)(L/mg)^1/*n*^, respectively (Figure 5b). For simultaneous PFOA adsorption, we measured RE(PFOA) = 98.1%, 88.1%, 80.9%, 84.5%, and 21.7% at [PFOA] = 10, 30, 50, 200, and 400 ppm, respectively. The adsorption capacity for PFOA was calculated to be *Q*_max_ = 134.6 mg/g and *K*_F_ = 31.08 (mg/g)(L/mg)^1/*n*^ using Langmuir and Freundlich models, respectively (Figure 5c and Table S7). The isotherm data indicated a superior fit to the Langmuir model compared to the Freundlich model, suggesting that monolayer surface adsorption is the primary mechanism for the removal of PS-MP and PFOA from aqueous solutions.^67^

Both MP and PFOA possess carboxylate functionalities and negative charges, leading to competitive surface adsorption on the granular CNN adsorbents. Given that microplastic beads possess a high density of pendent carboxylic acids, they likely adsorb more effectively to CNN adsorbents than PFOA due to multivalent interactions, which is supported by the results. Comparatively, the Langmuir model predicts an adsorption capacity for CNN4 using PFOA alone of *Q*_max_ = 146.8 mg/g (Figure S16), showing that competitive, simultaneous removal with PS-MPs only slightly affects the removal capabilities. This is hypothesized to be due to the penetration capabilities of PFOA allowing these contaminants to access pore sights within CNN structures that PS-MPs are incapable of filling due to their size. Overall, the adsorption capacity of CNN4 materials toward PFOA is similar to other gold-standard materials in the literature.^34,68^ This research underscores the potential of CNN gels as effective materials for addressing water contamination challenges posed by POPs such as MPs and PFAS via simultaneous removal. Follow up studies are currently underway expanding the studied PFAS and MP contaminants to include other derivatives commonly found in the environment.

To complement our study, a comparative analysis of microplastic removal techniques from previous studies was conducted, focusing on key parameters such as removal efficiency, reusability, and adsorption capacity, with the results summarized in Table S8. Following an extensive review of various methods, it was observed that the polymeric CNN system developed in this study exhibits notable advantages. These include a record-high adsorption capacity and effective adsorption characteristics across a wide pH range, particularly in basic environments such as seawater. Unlike many existing materials that are constrained by pH conditions or the size of pollutants, the CNN system demonstrates exceptional versatility and efficiency in the PS-MP, PS-NP, and simultaneous removal of PS-MP and PFAS. This innovative system offers a robust and highly effective solution for environmental remediation, addressing critical contamination challenges with superior performance.

### Adsorption Mechanism Studies

**Figure 6:**
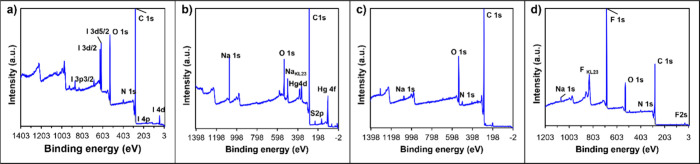
Full elemental survey XPS spectra for CNN4 (a), PS-MP (b), CNN4 after PS-MP adsorption (combined samples from [PS-MP] = 100–500 ppm) (c), and CN4 after simultaneous PS-MP and PFOA adsorption (combined samples from [PS-MP] = [PFOA] = 100–500 ppm) (d).

To study the adsorption mechanisms between CNN and MP/PFOA, the structures of CNN4, NN5, MP, CNN4 after adsorption of MP, and CNN4 after adsorption of MP and PFOA were analyzed using X-ray Photoelectron Spectroscopy (XPS). A general survey of these systems via XPS provided an understanding of the elemental states present in each system. The full XPS spectrum of CNN4 (Figure 6a) shows the presence of carbon (C), nitrogen (N), oxygen (O), and iodine(I). The iodine signal arises from methyl iodide used during the quaternization reaction and the iodide counterion expected for all quaternary amine centers (Figure S17). By comparing the chemical composition of CNN4 with that of NN5 (Figure S18), it is clear that NN5 consists only of C and O. The presence of the N 1s peak and iodine in CNN4 confirms the successful synthesis and functionalization of CNN4. The primary signal in the XPS spectrum of PS-MP is the surface carboxylic acid functionality and aromatic C_sp2_ peaks as expected (Figure 6b). The spectrum reveals C, O, sulfur (S), sodium (Na), and mercury (Hg) peaks, with S, Na, and Hg originating from thimerosal, a preservative used in the PS-MP sample (Figure S19). The XPS spectrum of CNN4 after adsorbing PS-MP at high concentrations (combination of samples exposed to [PS-MP] = 100 to 500 ppm) displays C, O, N, and Na, with the notable absence of iodine, suggesting that MP adsorbed onto the surface of CNN4 via an ion exchange mechanism (Figure 6c). The reduction in the intensity of the N signal compared to CNN4 before adsorption further indicates the adsorption process. Na is associated with the MP solution, while Hg and S are not observed, indicating that they might be removed during the washing steps. (Figure 6d) illustrates the XPS spectrum of CNN4 after the adsorption of both MP and PFOA. The sharp peak for fluorine (F) serves as a clear indicator of PFOA adsorption, confirming the interaction between CNN4 and PFOA in the system and again the absence of Iodine is the indication of ion exchange.

**Figure 7:**
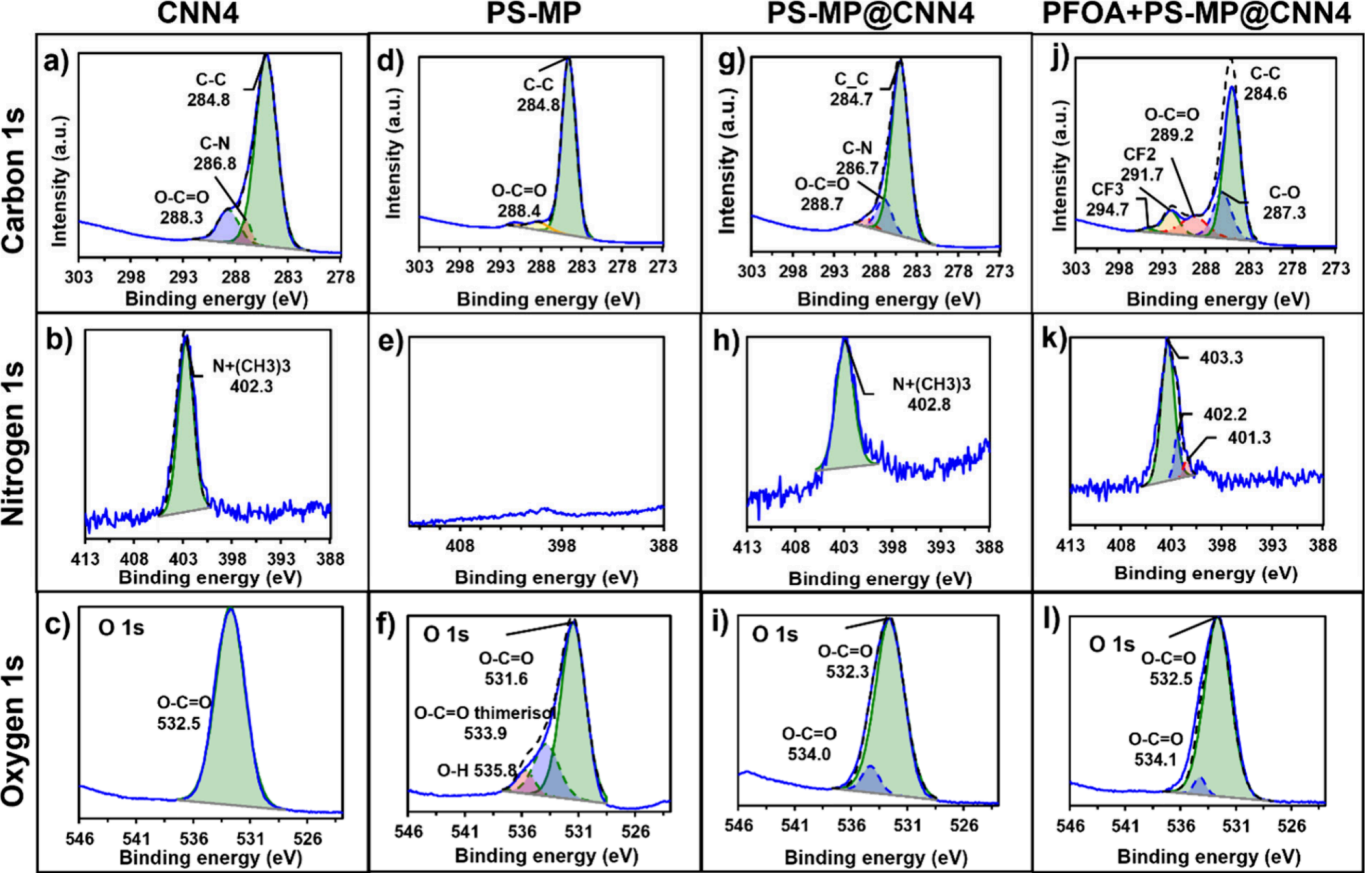
Deconvolution of high-resolution XPS spectra for CNN4 alone (a–c), PS-MPs alone (d–f), CNN4 after PS-MP adsorption (g–i; combined from adsorption experiments with [PS-MP] = 100–500 ppm), and CNN4 after simultaneous PS-MP and PFOA adsorption (j–l).

High-resolution XPS and attenuated total reflectance- infrared (ATR-IR) spectroscopy were employed to investigate the adsorption mechanisms for PFOA and PS-MPs. Prior to analysis, the XPS spectra were calibrated with the C 1s peak at 284.8 eV. The deconvolution of the C 1s, N 1s, and O 1s spectra for CNN4 (Figures 7a–7c), MP (Figures 7d–7f), CNN4 after MP adsorption (Figures 7g–7i), and CNN4 after the adsorption of both MP and PFOA (Figures 7j–7l) provide key evidence for the proposed ion-exchange adsorption mechanism^70^ For CNN4 the deconvolution analysis of C 1s (Figure 7a), resulted in three component signals ascribed to C–C (284.8 eV), C–N (286.8 eV), C=O (288.3 eV attributed to the ester group of the methacrylate polymeric backbone).^71^ In the case of PS-MP (Figure 7d), no C–N peak is observed as expected, showing only C–C (284.8 eV) and C=O (288.4 eV), which are characteristic of the carboxylic acid group functionality of PS-MP. After PS-MP adsorption onto CNN4 surfaces (Figure 7g), no significant change was observed in the C–C peak attributed to the similar functional groups present in both systems. However, the binding energy of C–N (286.7 eV) decreased, and the C=O peak (288.7 eV) shifted noticeably to higher binding energies. This suggests that both the C–N species in the quaternary ammonium pendants and the C=O species in the carboxylate groups participate in the adsorption of MP.^72^ Furthermore, upon adsorption of both MP and PFOA onto CNN4 (Figure 7j), the C–C signal remained largely unchanged, but the C=O peak (289.2 eV) shifted to even higher binding energies. New peaks corresponding to C–O (287.3 eV), CF2 (291.7 eV), and CF3 (294.7 eV) emerged, confirming the adsorption of PFOA and MP.

The high-resolution F 1s spectrum (Figure S20) was also analyzed to gain further insights into the PFOA adsorption mechanism. The binding energy for the F 1s peak of pristine PFOA was 689.0 eV.^73,74^ After adsorption, no shift in the F 1s binding energy was observed, suggesting that adsorption is not driven by any interactions between the C–F chain of PFOA and polymer backbone, which is expected due to the lack of fluorous character in the CNN microstructure. This indicates that electrostatic interactions and ion exchange are the dominant mechanisms in the adsorption process.

In the N 1s region, CNN4 exhibited a peak at 402.3 eV, which is characteristic of the quaternary ammonium group (R-N(CH_3_)_3_^+^) (Figure 7b).^69^ No N 1s peak was detected for MP (Figure 7e) as expected. After adsorption of MP onto CNN4 (Figure 7h), the peak corresponding to the quaternary ammonium group shifted to higher binding energies (402.8 eV), accompanied by a decrease in the intensity of the R-N(CH_3_)_3_^+^ peak. This suggests that the quaternary ammonium groups play an active role in the adsorption of MP by donating electron density.^69^ A similar trend was observed after the simultaneous adsorption of MP and PFOA onto CNN4 (Figure 7k), where the R-N(CH_3_)_3_^+^peak shifted further to higher binding energy (403.3 eV), and additional peaks in the range of 402.2–401.3 eV were attributed to interactions between PFOA, MP and CNN4.

For the O 1s region, the deconvolution of the CNN4 spectrum revealed a peak at 532.5 eV, corresponding to the ester group (O–C=O) of the polymer backbone (Figure 7c). In the MP spectrum (Figure 7f), two distinct C=O peaks were observed. One peak at 531.6 eV corresponded to the carboxylic acid group of MP, while another peak at 533.9 eV was assigned to thimerosal, the preservative. The peak at 535.8 eV was attributed to O–H groups and residual water.^75^ After the adsorption of MP and PFOA, the ester group C=O peak at 532.5 eV remained unchanged (Figures 7i–7l). However, the C=O peak corresponding to the carboxylate groups of MP and PFOA shifted to higher binding energies (534.1 eV), indicating the involvement of these functional groups in the adsorption process.

To further investigate the adsorption mechanism, FTIR-ATR analysis was conducted (Figures S21 and S22). The MP spectrum (Figure S21a) shows a peak at 1600 cm^–1^, which corresponds to the C=C stretching of the aromatic ring, as well as peaks at 2919 cm^–1^ (C–C stretching) and 3023 cm^–1^ (C–H stretching) (Figure S22a). The FTIR spectrum of CNN4 (Figure S21b) reveals characteristic peaks corresponding to the C–N stretching vibration of the quaternary ammonium group (R-N(CH_3_)_3_^+^) and the CH bending vibration of the ammonium methyl groups associated with the DAEMA monomer, observed at 962 cm^–1^ and 1455 cm^–1^, respectively.^76−78^ Additionally, bands at 1723 cm^–1^ and 1685 cm^–1^ are attributed to the carbonyl (C=O) group in the DAEMA monomer and the overlapping carbonyl groups (C=O) of the cross-linker.^79^ Furthermore, the peaks at 1137 cm^–1^ and 1058 cm^–1^ correspond to the asymmetric and symmetric stretching of the C–OC– bond in DAEMA and BMA.^80^ After the adsorption of MP, the characteristic peaks of MP are clearly observed in the CNN4 spectrum, confirming the successful adsorption (Figures S21, S22c, and S22d). To further elucidate the adsorption mechanism, notable shifts in the characteristic peaks for C–N stretching (R-N(CH_3_)_3_^+^) and CH bending of ammonium methyl groups, originally observed at 962 cm^–1^ and 1455 cm^–1^, respectively, were detected, shifting to 985 cm^–1^ and 1471 cm^–1^ following MP adsorption. These shifts are indicative of electrostatic interactions between the quaternary ammonium groups of CNN4 and the MP molecules. A similar trend is observed for PFOA molecules after adsorption, further supporting the involvement of electrostatic interactions in the adsorption process.^72^

### Reusability of Adsorbent

To assess the sustainability of CNNs, gel CNN4 was selected for multiple adsorption–desorption cycles due to its high capacity and performance. The interaction between the adsorbent surface, which contains quaternary amines, and the microplastics (MP) with carboxylic acid groups can be disrupted by charge screening. A salt wash, proved to be an effective method for regenerating the gel and ensuring its reusability.^34,69,72^ After the first adsorption cycle with 100 ppm MP, CNN4 was washed with 1 M NaCl for eight cycles, followed by eight washes with DI water, and finally rinsed with methanol (MeOH) to aid in dying. This process was repeated for three cycles, with only 8.9% of the gel’s original adsorption capacity lost (Figure S23a). This minor reduction is likely due to filtration losses and the strong interactions between the CNN4 and the MP. Despite this, CNN4 maintained a high adsorption capacity, indicating its potential for sustainable reuse. Various salts, including NaCl and ammonium acetate, were tested at different concentrations for desorption. Among them, NaCl was found to be the most effective, with higher concentrations yielding better results (Figure S15) The appearance of CNN4 before and after regeneration, under both room light and UV light, showed color changes, due to the desorption of MP and no physical changes on the gel supporting the gel’s durability across multiple cycles (Figure S23b).

## Conclusion

The described methodology of CNN formation used combined PhotoATR-PISA and photocuring to provide simple and unique syntheses of chemically- and morphologically tunable polymer nanoparticle networks for diverse applications in environmental remediation. In the present study, the synthesized CNN materials were examined for the adsorptive removal of PS-MP/NP mimics driven by electrostatic complexation of anionic MP/NP and positively charged CNN surfaces. Our best performing material, CNN4(Q_75_B_300_) demonstrated record-high adsorptive capacities for PS-MPs in tap water with remarkable *Q*_max_ = 1865 mg/g and *K*_F_ = 58.1 (mg/g)(L/mg)^1/*n*^ based on Langmuir and Freundlich isotherm models, respectively. This material also displayed impressive adsorption characteristics for PS-MPs in seawater, PS-NPs in tap water and PFOA in tap water with *Q*_max_ = 822, 318, and 146 mg/g, respectively, demonstrating the versatility of the material. Finally, CNN4 was also implemented for the simultaneous adsorption of PS-MPs and PFOA which, as expected, attenuated the overall adsorption capacity of each (ca. *Q*_max_(MP) = 478.8 mg/g, *Q*_max_(PFOA) = 134.6 mg/g) but still provided impressive performance enabling potential dual-use applications. The ion-exchange adsorption mechanism was further characterized and confirmed using combinations of XPS and ATR-FTIR spectroscopy.

## Supporting Information Available

Instrumentation used, synthesis and characterization of all materials, adsorption characterization with PS-MP/NP and PFOA, adsorption mechanism studies, reusability studies of CNN adsorbents, and comparative studies of microplastic removal techniques (PDF)
