# Supplementary material for: Cationic Nanoparticle Networks (CNNs) with Remarkably Efficient, Simultaneous Adsorption of Microplastics and PFAS
Source: ACS Appl Mater Interfaces. 2025 Feb 10;17(7):10732–44. doi: 10.1021/acsami.4c21249 (PMC11843541; doi:10.1021/acsami.4c21249)
Supplement: Supplementary file 1 — am4c21249_si_001.pdf [file am4c21249_si_001.pdf]

Supporting Information

Cationic Nanoparticle Networks (CNNs) with Remarkably Efficient, Simultaneous Adsorption of Microplastics and PFAS

Shayesteh Tafazoli,[a] Dylan B. Shuster,[a] Ali Shahrokhinia,[a,b] Sahaj Rijal,[a] Dorcas M. Ruhamya,[a] Kamryn A. Dubray,[a] David J. Morefield,[c] and James F. Reuther*[a]

[a] Department of Chemistry, University of Massachusetts Lowell, Lowell, MA 01854, United States
[b] BASF Corporation, AMIC, DZ3, 1609 Biddle Avenue, Michigan 48192 Wyandotte, USA
[c] Department of Chemistry, Johns Hopkins University, Baltimore, MD 21218, United States
Corresponding Author email: James_Reuther@uml.edu

Table of Contents

1. **Instrumentation**

2. **Synthesis and characterization**

3. **Characterization of PISA nanoparticles and CNN adsorbents**

4. **Adsorption characterization with PS-MP/NP and PFOA**

5. **Adsorption mechanism investigations**

6. **Reusability of CNN adsorbent**

7. **Comparative studies of microplastic removal techniques**

8. **References**

1. Instrumentation

1.1. NMR spectroscopy

The ^1H and ^{13}C nuclear magnetic resonance (NMR) spectra were obtained on a JEOL ECZ 400 MHz spectrometer, employing $CDCl_3$ and DMSO-d_6 (Sigma-Aldrich) as solvents and referencing spectra to residual solvent signals. The ^1H NMR spectra were acquired with an average of 16 scans.

1.2. Gel permeation chromatography (GPC)

The molecular weight (M_n) and molecular weight distribution ($Đ$, M_w/M_n) were determined by employing two different GPC machines to get the better resolution, Tosoh HLC8320 EcoSEC GPC system and Waters Alliance 2695 HPLC system. Tosoh HLC8320 EcoSEC GPC system was equipped with a refractive index (RI) detector, operating at a temperature of 40 °C. The styragel column (TSKgel SuperH4000) were calibrated using monodispersed polystyrene standards and HPLC-grade tetrahydrofuran (THF) was used as the eluent at a rate of 0.5 mL/min. All chromatographic runs had a duration of 10 minutes.

Waters Alliance 2690 HPLC system with 2414 Refractive index detector. We use Waters Styragel HR columns 1,3, and 4 for molecular weight determination. This system is run using 100% tetrahydrofuran(THF only) as the mobile phase at a flow rate of 0.9ml/min. Each sample must be dissolved in 100% tetrahydrofuran for 24 hours before analysis at 10 to 20 mg/ml, and filtered through a 0.45 micron nylon or PTFE filter. Polystyrene standards from a molecular weight of 500 to 400K are used to create the calibration curve. Each GPC run takes 50 minutes to complete. Empower 2 is the software system for controlling the separation and analysis.

1.3. Transmission Electron Microscopy (TEM)

Transmission electron microscopy (TEM) was conducted using a Philips CM12 electron microscope operating at 120 kV. The PISA nanoparticle dispersions were diluted with methanol to concentration = 1.0 mg/mL, and the resulting dispersions were drop-casted onto carbon-coated copper grids (Ted Pella). Subsequently, the grids were dried in a vacuum oven. The TEM imaging of all samples was performed without the use of any external staining agents.

1.4. Dynamic light scattering (DLS)

To determine the average hydrodynamic diameters and size distribution of PS-MPs and NPs, dynamic light scattering (DLS) was performed using a Horiba SZ-100 Particle Analyzer. The scattered light was detected at an angle of 90°. The samples were prepared by diluting nanoplastics suspension in deionized (DI) water to achieve various concentrations for the calibration curve and testing. The solutions were subsequently filtered using a 1 µm filter and maintained at a temperature of 25 °C.Data processing was carried out utilizing the general-purpose algorithms available in the SZ-100 Software. To ensure accuracy, triple runs were conducted for each sample, and the results were reported as an average. The zeta potential of microplastics in aqueous suspension was determined through a Zetasizer Horiba SZ-100.

1.5. Fluorescence spectroscopy

Yellow-green fluorophore-labeled polystyrene (PS) microparticles (diameter = 1.0 µm) with carboxylic acid functionality, used as fluorescent microplastic mimics, were procured from Invitrogen by Thermo Fisher. These PS particles possessed excitation and emission wavelengths

of 505 nm and 515 nm, respectively. To quantify the concentration of fluorescent PS microplastic mimics (d = 1.0 μm), the fluorescence spectra of the microplastic suspensions were measured at various concentrations using a JASCO FP-8500 fluorescence spectrophotometer. The measurements were conducted at ambient temperature. Prior to conducting tests on unknowns, a range of microplastic suspensions were measured, spanning from 10^{-3} mg/L to 50 mg/L, to establish a standard calibration curve.

1.6. Brunauer-Emmett-Teller (BET) Surface Area Analysis

The specific surface area, pore volume, and pore size distribution of polymeric nanoparticle network samples were estimated with N_2 adsorption-desorption tests using a Micromeritics ASAP2020 Plus Automatic Micropore and Chemisorption Analyzer and the samples were degassed at 100 °C and 55°C, each for 20 h. The surface area (SBET) was estimated by Brunauer-Emmett-Teller (BET) method and the pore size distribution was determined by Density Functional Theory (DFT). The total pore volume (VT) was obtained using the adsorbed nitrogen at a relative pressure p/p_0 of approximately 0.99. The pore diameter was calculated using software that utilizes the Isotherm Tabular Report to determine the pore volume first. The pore diameter was then derived using the formula: Pore Diameter = (Pore Volume × 4) / Surface Area

1.7. Quadrupole Time-of-Flight Mass Spectrometer (Q-TOF)

Waters Xevo G2-XS Tandem quadrupole time-of-flight (Q-TOF) mass spectrometer with Acquity UPLC and TUV detector for separation of complex mixtures of small molecules to large molecules. The Xevo G2-XS Q-TOFcombines StepWave ion optics for robust sensitivity with the XS collision cell to achieve low levels of sensitivity with accurate mass detection. It can measure a m/z ratio of up to 8000. Ionization modes include electrospray interface (ESI). PFOA was screened in negative ion mode (m/z 412.96791) with mobile Phase(s): Methanol, Ammonium Acetate Buffer (2 mM); gradient method.

1.8. X-Ray Photoelectron Spectroscopy

Surface analysis was conducted using a Kratos Axis Supra XPS system (Kratos Analytical, 2017). The system operates under a vacuum of 10^{-9} Torr or better to ensure high-quality measurements. It features a 165 mm mean radius hemispherical analyzer coupled with a Spherical Mirror Analyzer, enabling sensitive analysis and fast parallel imaging with the 2D delay line detector. The magnetic immersion lens provides high sensitivity on small areas, with spatial resolution as fine as 1 μm, allowing for precise analysis of samples down to 15 μm.

The charge neutralization system, equipped with either a Mg/Al or Al/Ag monochromatic source, enables the acquisition of high-resolution spectra from insulating materials like polymers. This capability is crucial for analyzing samples that do not easily conduct electricity.

For this experiment, polymeric powder samples including (CNN4, PS-MA, PS-MA@CNN4, and PFOA + PS-MA@CNN4) were dried in a vacuum oven prior to analysis to prevent any degassing during the measurement. Since the detection limit of the system is approximately 500 ppm, all samples were prepared with similar concentrations within this range. The powder samples (20-30 mg) were mounted on carbon tape using a plane dual-height sample holder for optimal positioning during analysis.

1.9. FT-IR Spectroscopy

FT-IR spectra obtained with a Jasco FT/IR-6600 (100–4000 cm^{-1}) with ATR apparatus were used for structural characterization of CNN, MP and PFOA before and after adsorption.

2. Synthesis of Macroinitiators, PISA nanoparticles and CNN adsorbents

2.2. Preparation of Macroinitiators

2.2.1. Synthesis of PDAEMA(D)-based macroinitiator

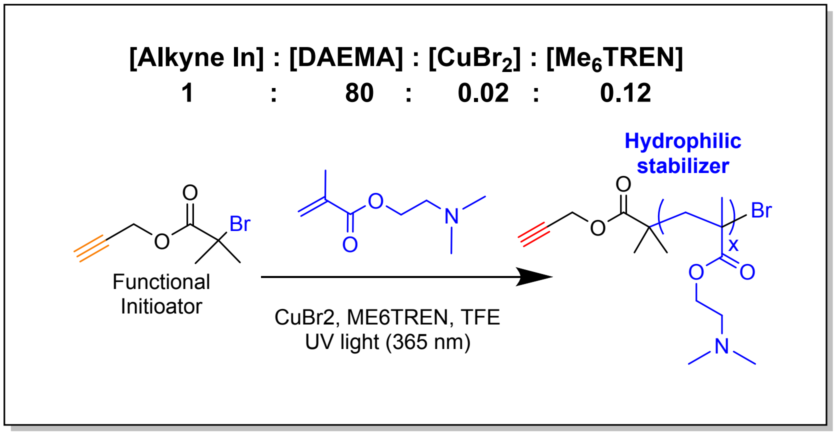

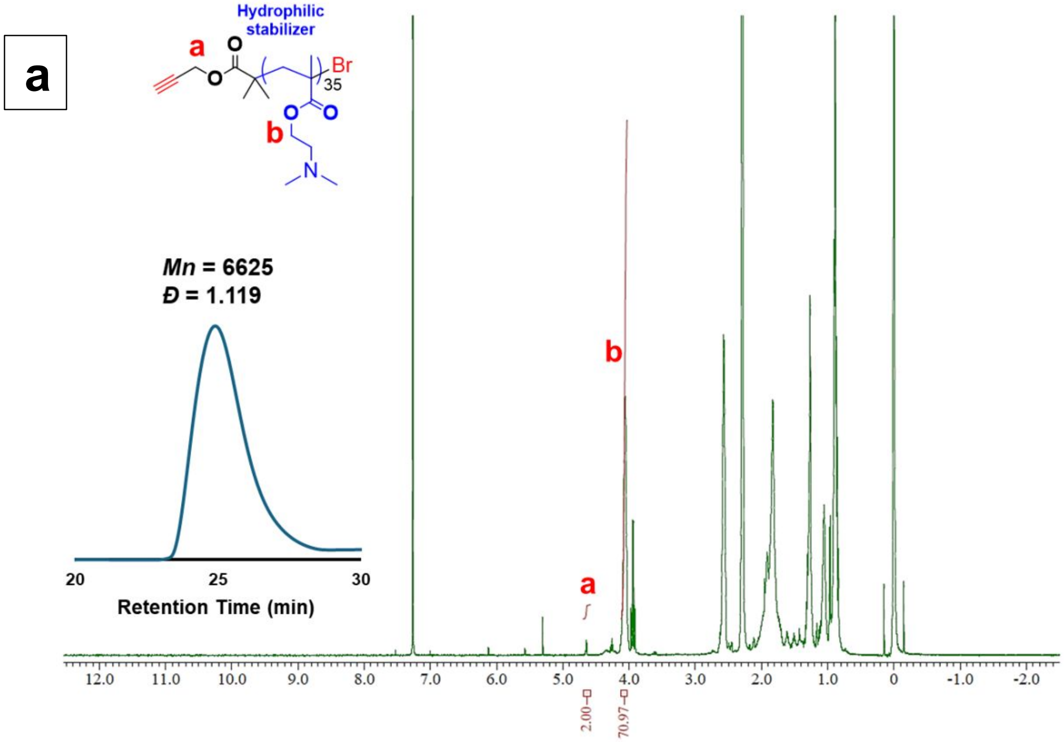

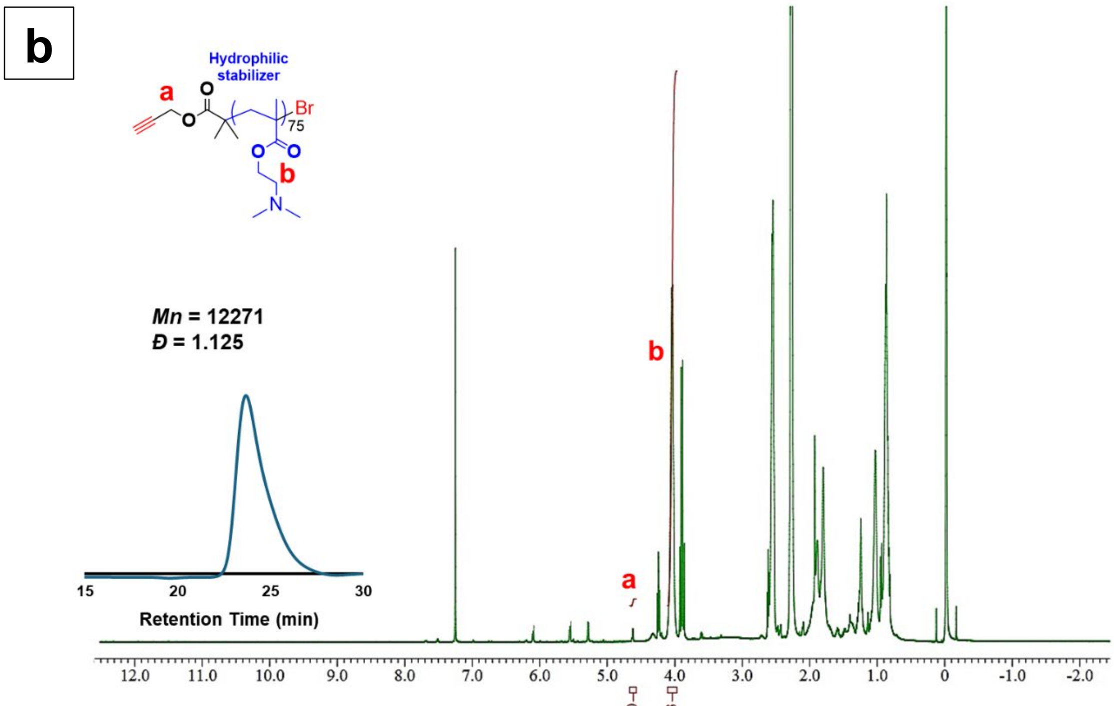

Figure S1. ^{1}H NMR spectra and GPC analysis of D-based macroinitiators with targeted degrees of polymerization DP = 40 (a) and DP = 80 (b). Peaks used for calculations of DP and M_n are indicated.

2.2.2. Synthesis of POEGMA-based macroinitiator

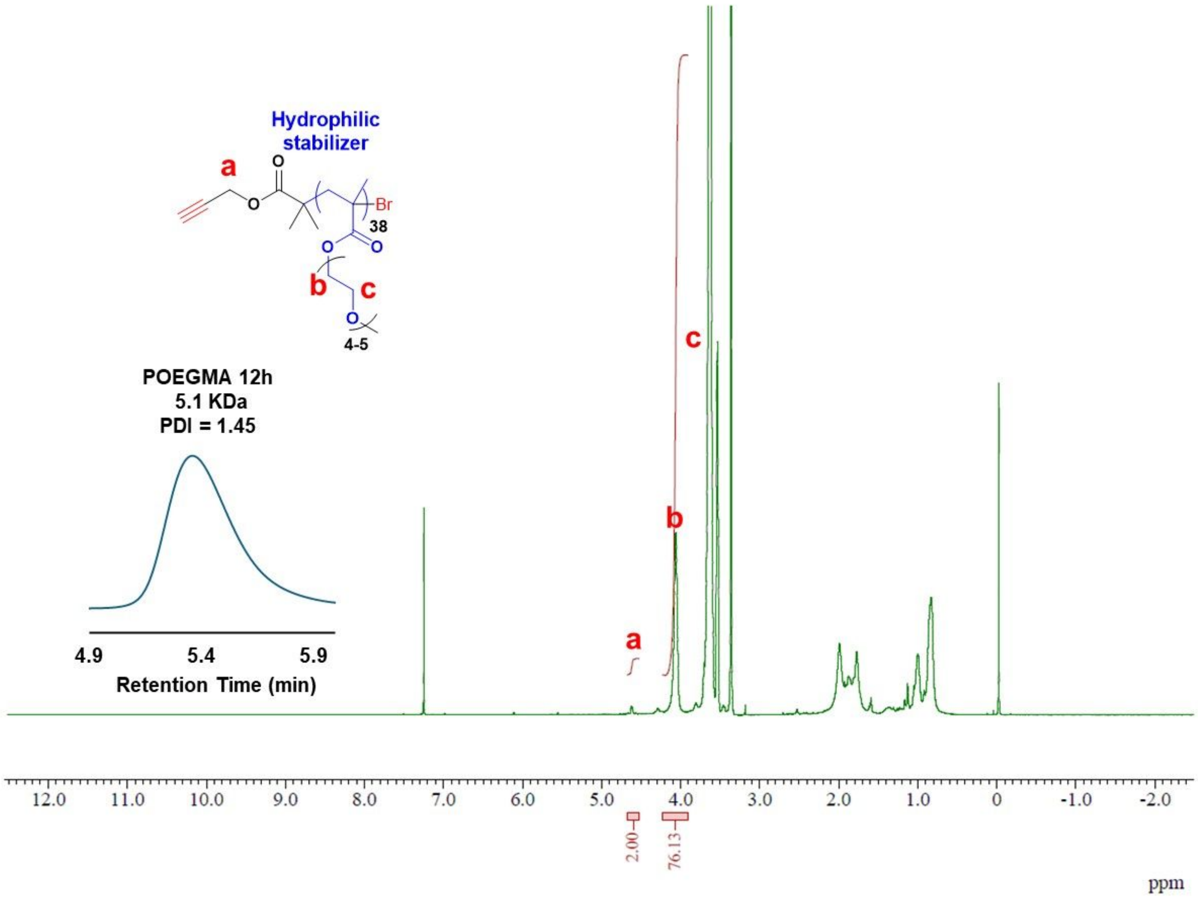

Figure S2. ^{1}H NMR spectra with peak assignments used for M_n calculations and GPC chromatograms for P-based macroinitiators used to synthesize non-ionic NN adsorbents.

2.3 Synthesis of Cationic Nanoparticle Networks (CNN) using two-pot PhotoATR-PISA and photo-curing

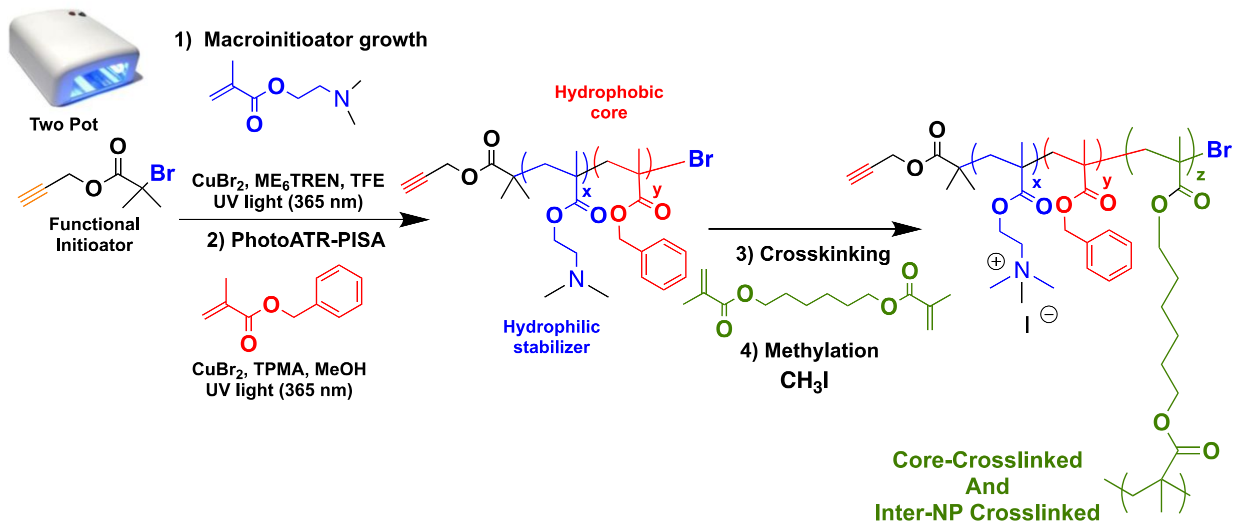

Scheme S1. Synthesis of CNN adsorbents using two-pot PhotoATR-PISA followed by photo-curing and post-polymerization methylation of amine pendant groups.

3. Characterization of PISA nanoparticles and CNN adsorbents

Prior to the addition of the crosslinker, the chemical structure of the polymeric nanoparticles was analyzed using ^{1}H NMR spectroscopy while the molecular weight of dissolved block copolymers was determined through GPC and ^{1}H NMR analysis. TEM was employed to visualize nanoparticle morphology prior to crosslinking. Furthermore, the surface morphologies of gel samples were examined using SEM. Prior to SEM testing, the samples were coated with a thin layer of gold via sputter-coating. BET surface area analysis was conducted to determine overall surface area and pore size distribution in all NN gels prior to methylation due to interference in analysis caused by permanently cationic CNN gels.

Table S1. PISA1 – 5 compositions determined using ^{1}H NMR and GPC characterization prior to crosslinking.

Sample	DP of M.In	Target DP for core (B)	M_n From GPC (kDa)	$Đ$ (M_w/ M_n)	DP(B) From GPC	DP(B) From ^{1}H NMR
PISA1	35	100	25.2	1.48	112	125
PISA2	75	100	29.0	1.76	95	88
PISA 3	35	300	49.6	2.23	250	220
PISA4	75	300	56.9	2.09	255	282
PISA5	37	100	25.3	1.45	114	98

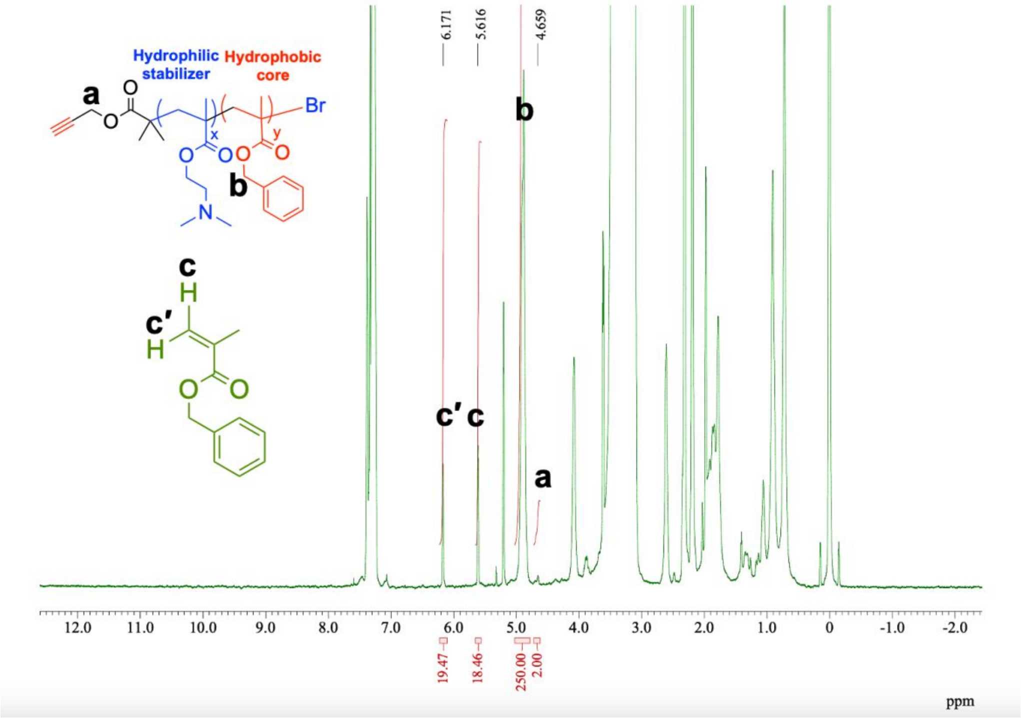

Figure S3. ^{1}H NMR analysis for PISA1 nanoparticles prior to crosslinking which revealed distinct peaks corresponding to the BMA monomer (labeled as c and c') and the polymerized PBMA (labeled as b) which can be integrated relative to each other to understand percent conversion.

Table S2. The average diameter of PISA1 – 5 nanoparticles prior to crosslinking and CNN1 – 4; NN5 nanoparticles after crosslinking as determined by TEM and SEM statistical analysis, respectively.

Sample	Average Particle Size (TEM)	Average Particle Size (SEM)
CNN1	46.60nm ± 6.99	83.76 nm ± 9.71
CNN2	149.3 nm ± 45.63	206.18 nm ± 36.95
CNN3	186.6 nm ±19.29	279.39 nm ± 50.39
CNN4	238.8 nm ±73.17	315.45 nm ± 70.71
NN5	106.3 nm±49.76	115.47 nm± 25.32

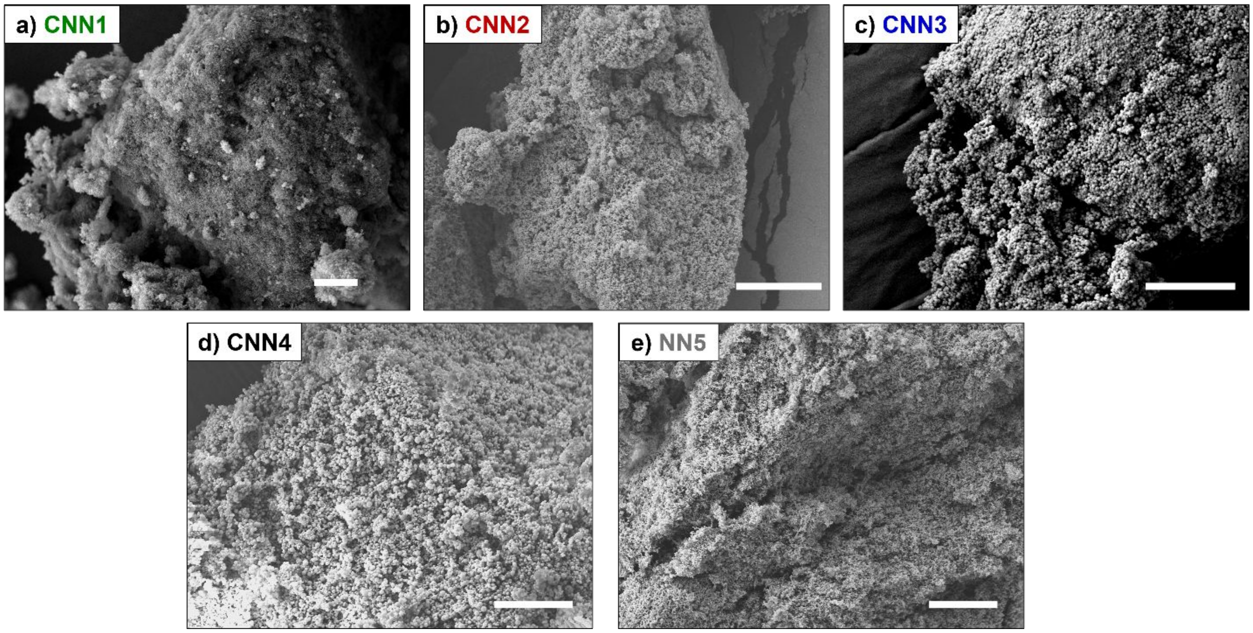

Figure S4. Zoomed out SEM images of CNN1 (a), CNN2 (b), CNN3 (c), CNN4 (d), and NN5 (e) demonstrating the uniformity across the sample (scale bar = 10.0 μm).

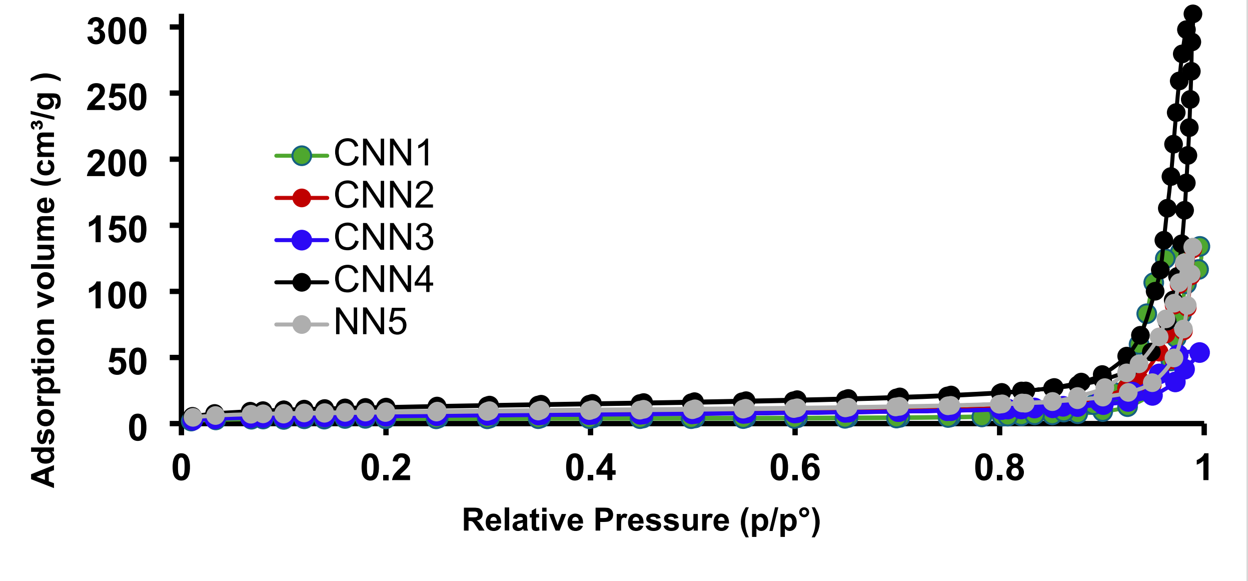

Figure S5. BET isotherm analysis performed on all samples prior to methylation and the formation of the cationic gel.

Table S3. BET surface area analysis of all CNNs, including total pore volume and average pore diameter measurements from adsorption studies. The pore diameter was calculated using software that utilizes the Isotherm Tabular Report to determine the pore volume. The highest quantity of adsorbed nitrogen gas (cm^3/g STP) prior to desorption was divided by 647 (a constant for converting liquefied nitrogen volume to gas volume at 77 K) to calculate the pore volume. The pore diameter was then derived using the formula: Pore Diameter = (Pore Volume × 4) / Surface Area.[2-3]

Sample	Surface Area (m^2/g)	Adsorption total pore volume of pores (cm^3/g)	Adsorption average pore diameter (nm)
CNN1	12.2093 ± 0.4261	0.163508	53.5685
CNN2	25.1620± 0.5112	0.204137	32.3022
CNN3	19.6492± 0.4095	0.076159	15.5440
CNN4	42.6361± 0.8419	0.479358	44.9720
NN5	27.0123± 0.5652	0.231273	34.2471

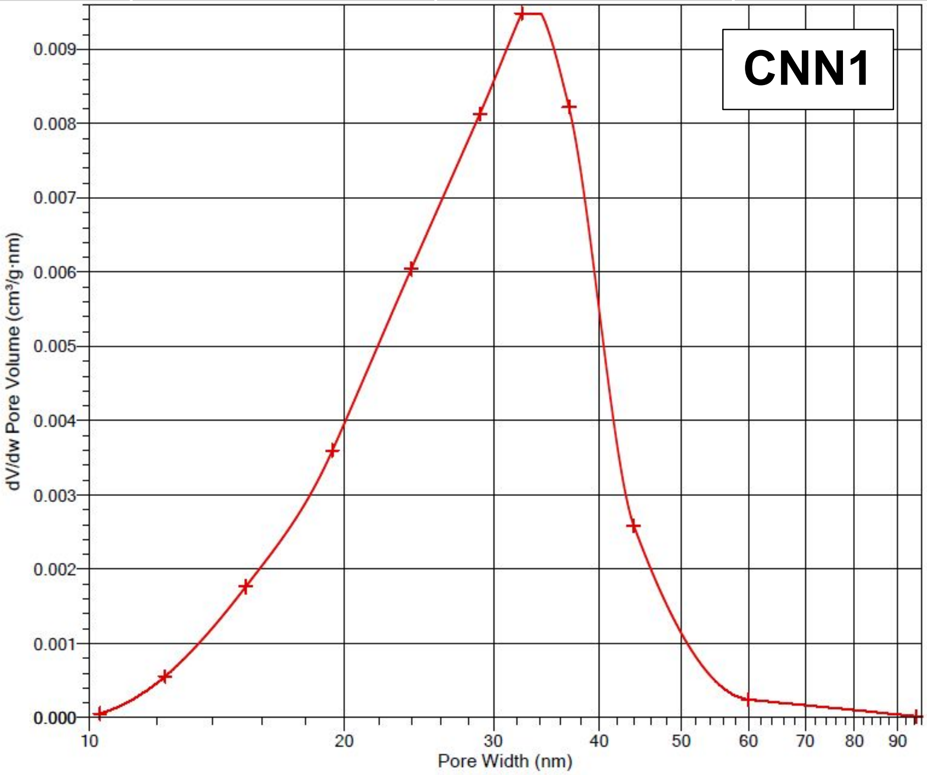

Figure S6. Pore-size distribution plot for CNN1

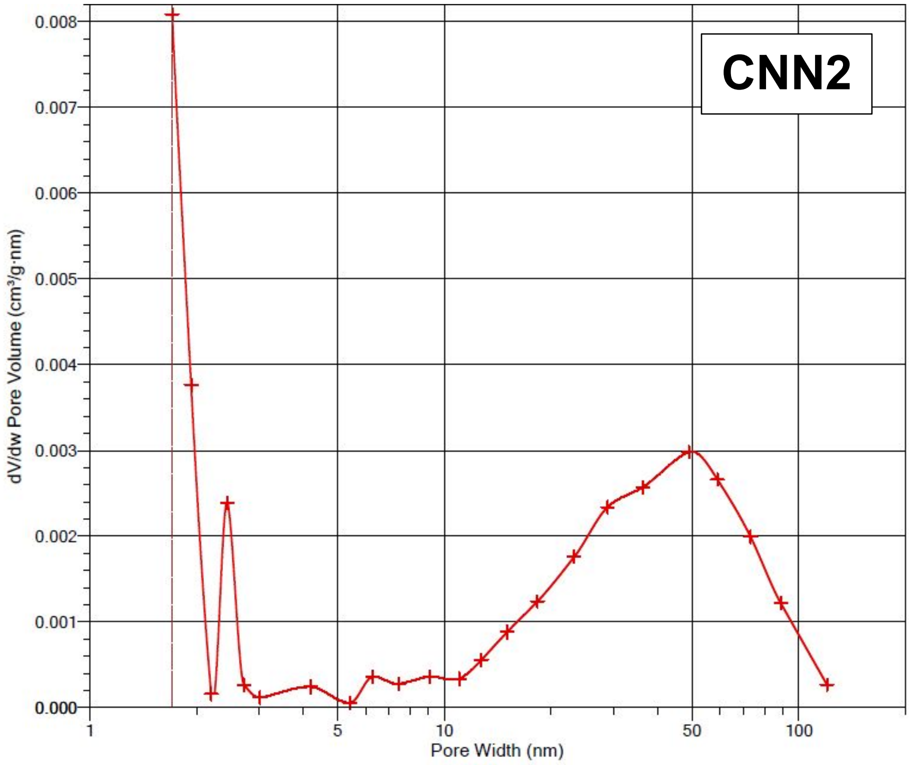

Figure S7. Pore-size distribution plot for CNN2

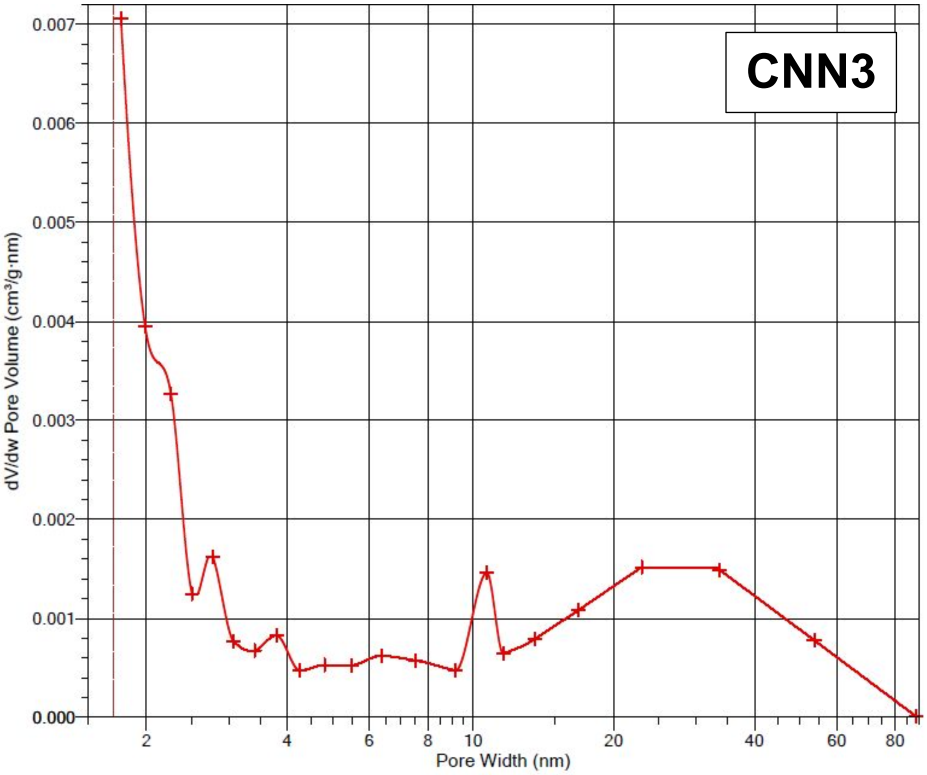

Figure S8. Pore-size distribution plot for CNN3

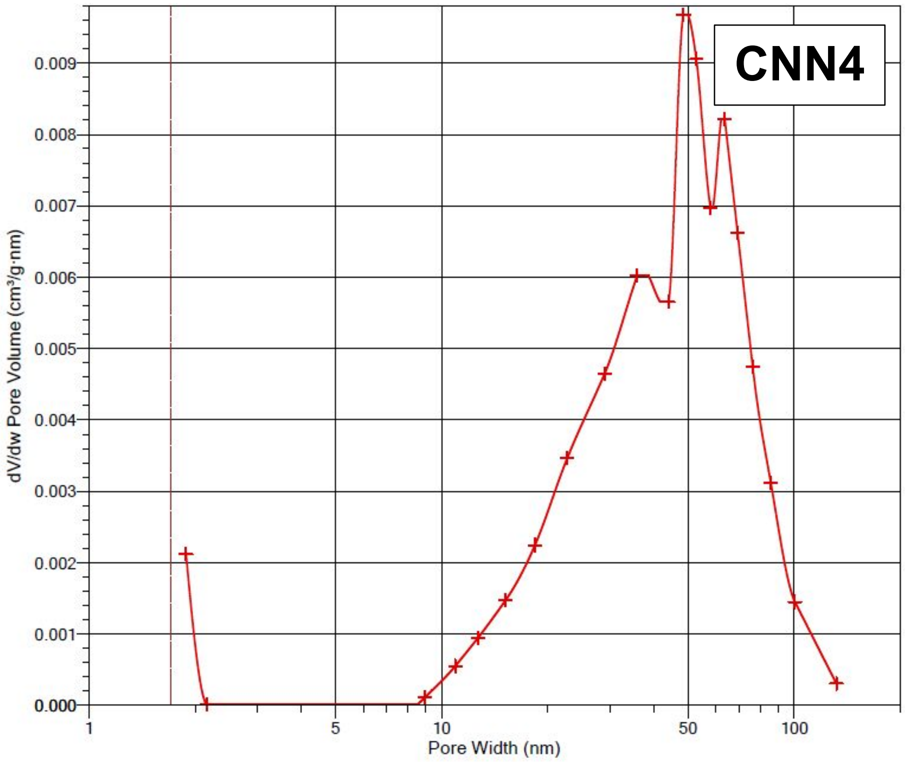

Figure S9. Pore-size distribution plot for CNN4

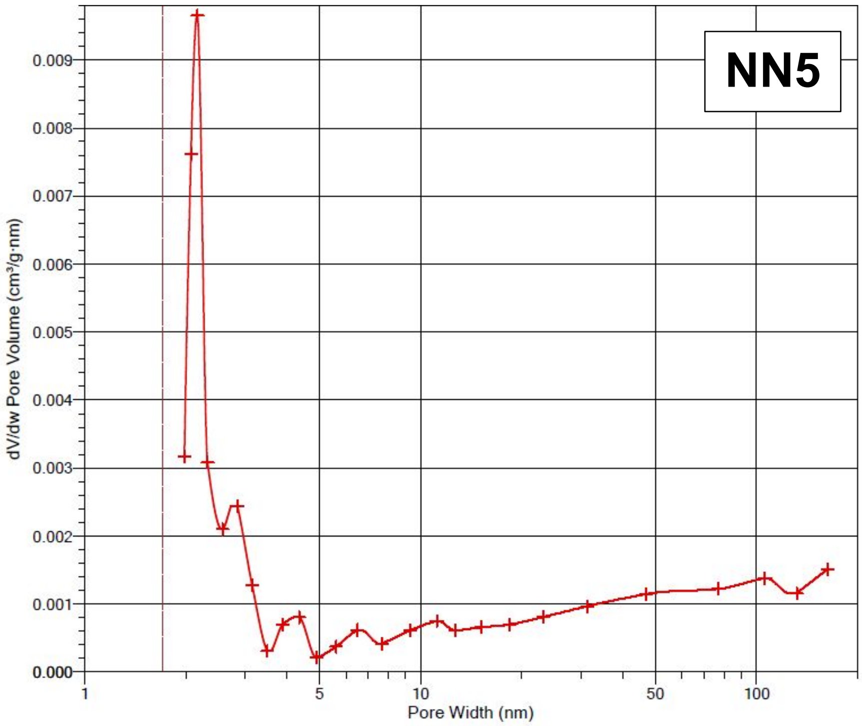

Figure S10. Pore-size distribution plot for NN5

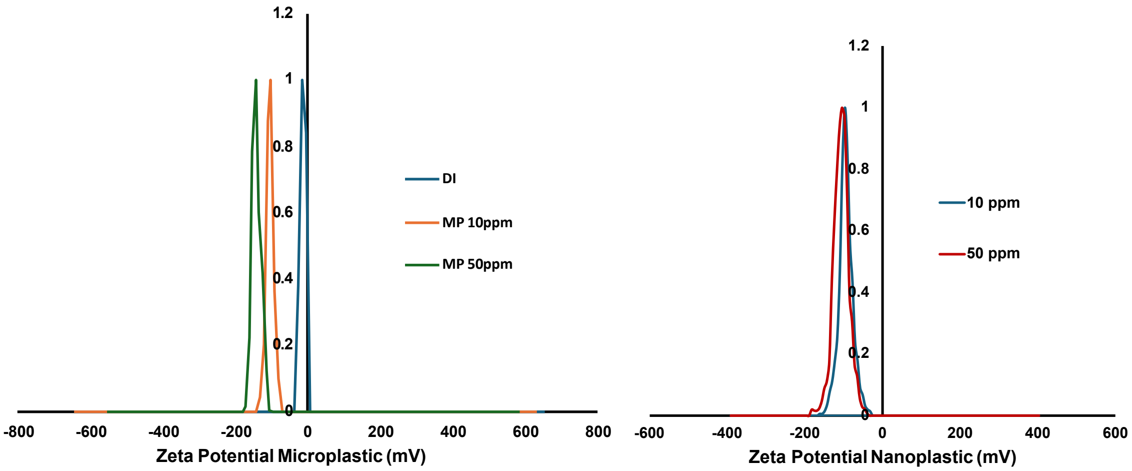

Figure S11. Zeta potential of PS-MPs and PS-NPs

Table S4. Zeta potential of PS-MPs and PS-NPs at various concentrations

Concentration (ppm)	Zeta Potential MP (mV)	Zeta Potential NP (mV)
10 ppm	-100.0	-95.8
50 ppm	-142.3	-104.5

4. Adsorption Characterization for PS-MP/NP and PFOA Contaminants

4.1. Microplastic removal experiment

For batch adsorption experiments, a stock solution of polystyrene microplastics (PS-MP; d = 1.0 μm) or nanoplastics (PS-NP; d = 100 nm) was prepared using deionized water. Subsequently, 10 mg of CNN or NN gel and 10 mL of the PS-MP/NP solution were added to a 20 mL glass scintillation vials. The vial was then subjected to rotational motion using a fixed-angle rotator, rotating at 40 rpm for a duration of 48 hours. In all adsorption experiments, one blank sample absent of CNN/NN adsorbents was made and used as reference control to exclude contributions of background adsorption (i.e., to glass walls of vials). Removal efficiencies (RE) were calculated based on this reference value. Following the incubation period, the polymeric adsorbents were separated from the solution through centrifugation at 3000 rpm for 5 minutes. It is important to note that the centrifuge speed and duration were optimized to solely remove gel particles without affecting the suspended PS-MP/NPs. Subsequently, the remaining concentration of microplastics in the solution was measured to determine the removal efficiency via fluorescence spectroscopy. The removal efficiency and adsorption capacity of the gel adsorbent samples were calculated using the equations provided below.

Equation S-1:

$$Removal\ efficiency\ (\%) = \frac{(C_0 - C_t)}{C_0} \times 100\ \%$$

Equation S-2:

$$Adsorption\ capacity\ (Q_e) = \frac{(C_0 - C_t) \cdot V}{W}$$

Where, C_0 and C_t are the concentration of the microplastic solution in reference control sample and with CNN adosrbent at t = 48 h, respectively. The adsorption capacity (Q_e in mg/g) is the mass of the microplastics (in mg) adsorbed by 1.0 g adsorbent. V and W are the volume of the solution and the mass loading of adsorbents used in each experiment, respectively.

Calibration curves for assessing removal efficiency and adsorption capacity

To quantify the concentration of fluorescent PS-MPs, the fluorescence spectra of the microplastic suspensions were measured at various concentrations using a fluorescence spectrometer. The fluorescence intensity exhibits a linear correlation with the concentration of microplastics in constructed calibration curves. Prior to conducting the tests on unknowns, a range of microplastic suspensions were measured, spanning from 10^{-4} mg/L to 50 mg/L, to establish a standard calibration curve (**Figure S11**).

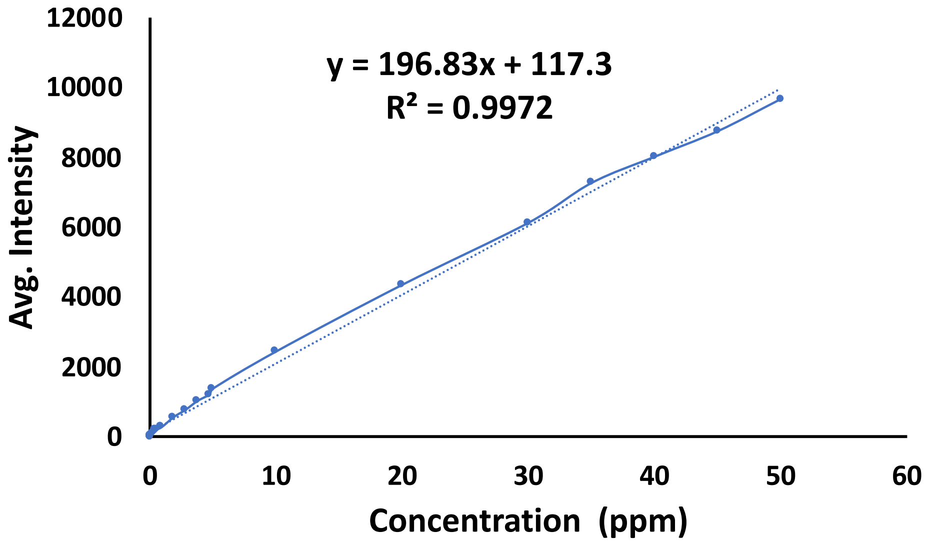

FigureS12. Calibration curve of fluorescent microplastic mimics (d = 1.0 μm) at different concentration obtained by fluorescence spectroscopy.

For non-fluorescent PS-NPs, dynamic light scattering (DLS) was employed for quantification of removal efficiency. DLS measurements were utilized to determine the concentration of nanoplastics in suspension by detecting the particles based on the amount of light scattered by the sample. These measurements are presented as "DLS derived count rate" in kilo-counts per second (kcps), which account for the observed count rates normalized to 0% attenuation at 100% laser power. Extensive analyses have demonstrated that this method provides the most reliable particle size identification via DLS, as DLS measurements are susceptible to potential "false positive" outcomes.[4] To ensure quantitative comparability across measurements, uniform measurement parameters were employed. The measurements consisted

of a total of three runs, each consisting of ten sub-runs. Each sub-run had a measurement duration of 10 seconds and was performed at a fixed angle of 90° and a temperature of 25 °C.

Reference measurements were conducted using pure, microfiltered water. Derived count rates were analyzed to determine the presence or absence of scattering centers and colloidal particles. Derived count rates below 2.0 x 10^3 kcps indicated the absence of scattering centers and colloidal particles, while truly colloidal reference samples exhibited derived count rates ranging from several hundreds to thousands kcps. The derived count rate served as a quality criterion to assess the presence or absence of colloids. Data processing was performed using the SZ-100 Software, utilizing the general-purpose algorithms available within the software for analysis. The DLS-derived count rate demonstrates a linear relationship with the concentration of nanoplastics. To establish a standard calibration curve, a series of PS- NP suspensions ranging from 0.1 mg/L to 33.3 mg/L were prepared prior to conducting the experiments (**Figure S12**).

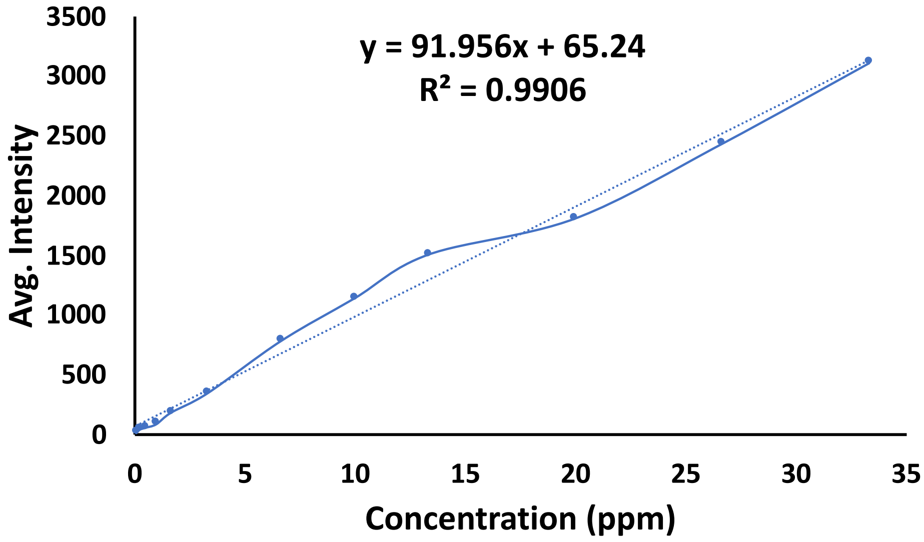

Figure S13. Calibration curve of non-fluorescent nanoplastic mimics (d = 100 nm) at different concentration obtained by DLS derived count rates.

4.2. Microplastic adsorption kinetics

The kinetics of MP adsorption were systematically analyzed and modeled. The removal process was effectively represented by pseudo-second order kinetic models, enabling the calculation of kinetic parameters according to **Equation S-3**:

$$\frac{t}{q_t} = \frac{1}{k_2 q_e^2} + \frac{t}{q_e}$$

Where **t** is the contact time (h), $\mathbf{q_t}$ (mg/g) and $\mathbf{q_e}$ (mg/g) represent the amounts of MPs adsorbed on the CNN at time (h) and equilibrium, respectively. $\mathbf{k_2}$ (g mg^{-1} min^{-1}) is the rate constant for Pseudo-second order kinetic model.

Table S5. Pseudo-second order kinetic models employed to derive and calculate the associated kinetic parameters for the adsorption process

Sample	**Pseudo-second order kinetic model**		
	k_2 (g mg^{-1} min^{-1})	q_e (mg/g)	R^2
NN5(P37-B100)	0.0474	12.45	0.9982
CNN1(Q35-B100)	0.0674	21.74	0.9993
NN2(D75-B100)	0.0620	32.15	0.9934
CNN2(Q75-B100)	0.0612	86.21	0.9995
CNN3(Q35-B300)	0.1220	82.64	1.000
CNN4(Q75-B300)	1.3520	96.15	1.000

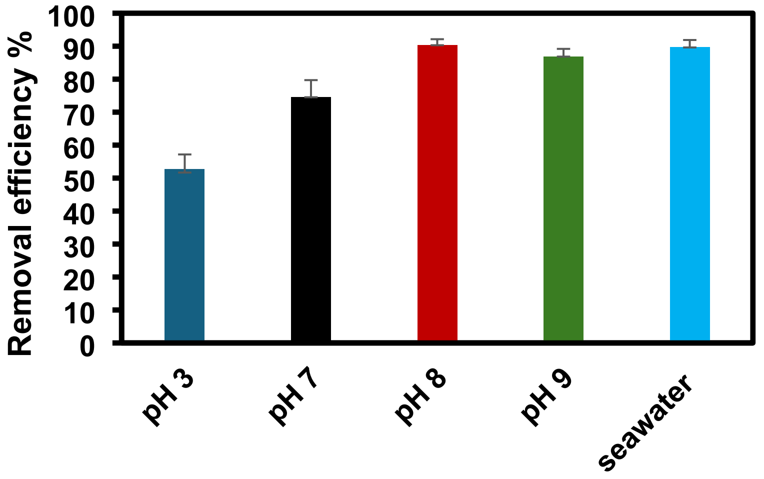

Figure S14. Removal efficiency of PS-MPs (c = 50 ppm) with CNN4($Q_{75}B_{300}$) at various pH and in simulated seawater, following the guidelines outlined in ASTM D1141-98(2021).

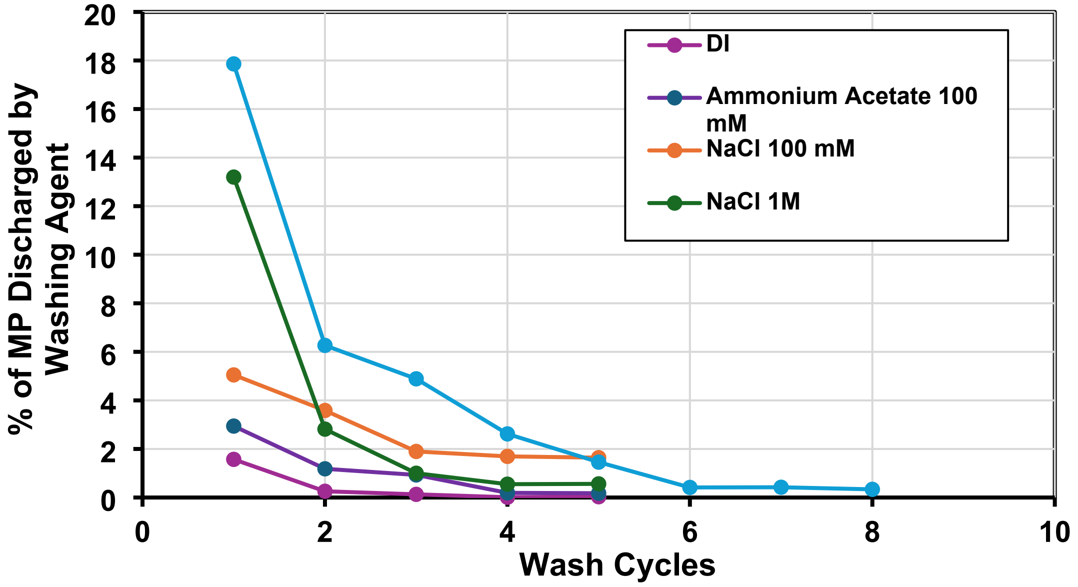

Figure S15. Washing of saturated CNN4($Q_{75}B_{300}$) adsorbents with deionized (DI) water, ammonium acetate (c = 100 mM), and sodium chloride (c = 100 mM, 1 M and 6 M).

4.3 Procedure and sample preparation for batch PFOA adsorption experiments:

For batch adsorption experiments, a stock solution of PS-MPs and PFOA was prepared using deionized water. Subsequently, 10 mg of CNN4 gel (best performing gel) was added to 10 mL of the microplastic-PFOA mixture The sample was then subjected to rotational motion using a fixed-angle rotator, operating at 40 rpm for a duration of 48 hours. Following the incubation period, the polymeric adsorbents were separated from the solution through centrifugation at 3000 rpm for 5 minutes. The remaining concentrations of microplastics and PFOA in the supernatant were then measured to determine the removal efficiency, compared to a control sample lacking adsorbent gels. For experiments with no PS-MP introduced, this step was omitted.

For each microplastic-PFOA sample, an initial dilution was performed by transferring 2 mL of the sample and filtering it through a 25 mm, 0.45 µm polypropylene membrane filter (VWR North America) to remove any microplastics into a 10-mL vial. To desorb any PFOA retained on the filter, 1.0 mL of methanol was subsequently filtered through, yielding a final sample volume of 3.0 mL.[3] LCMS vials (2 mL, Agilent USA) were prepared by diluting the 3-mL samples with water to achieve a concentration of 1 mg/L; if the sample concentration exceeded this limit, the samples were adjusted to 1 mg/L to prevent PFOA adsorption to the gel and to align with the calibration curve. The samples were then directly injected into the LCMS QTOF for characterization and quantification.

4.4 Adsorption isotherms

The equation for the Langmuir isotherm can be written as:

Equation S-4:

$$q_e = \frac{Q_{max} K_L C_e}{1 + K_L C_e}$$

where q_e is the amount of solute adsorbed per unit mass of sorbent (mg/g), C_e is the equilibrium concentration of the solute in the solution (mg/L), and K_L is the Langmuir adsorption constant (L/mg). The K_L value is related to the affinity of the solute for the sorbent surface.

The Freundlich equation can be expressed as:

Equation S-5:

$$q_e = K_F {C_e}^{1/n}$$

where q_e is the amount of solute adsorbed per unit mass of sorbent (mg/g), C_e is the equilibrium concentration of the solute in the solution (mg/L), K_F is the Freundlich constant related to adsorption capacity, and n is the Freundlich exponent that indicates the intensity of adsorption.

Table S6. All calculated values related to Langmuir and Freundlich models for all CNNs

**Gel**	**Langmuir**			**Freundlich**		
	**K_l (L/mg)**	**Q_{max} (mg/g)**	**R^2**	**K_f (mg/g)(L/mg)$^{1/n}$**	**n**	**R^2**
CNN1_$Q_{35}B_{100}$	0.002935	62.74	0.9384	1.296	1.856	0.9743
CNN2_$Q_{75}B_{100}$	0.00271	533.0	0.9903	3.293	1.324	0.9865
CNN3_$Q_{35}B_{300}$	0.01321	48.47	0.8273	1.658	1.619	0.8578
CNN4_$Q_{75}B_{300}$	0.007699	1865	0.9764	58.08	1.847	0.9855
CNN4_$Q_{75}B_{300}$ **PS-NP**	0.03152	318.4	0.9911	26.92	2.150	0.9758
CNN4_$Q_{75}B_{300}$ **Seawater**	0.009768	822.3	0.9959	14.37	1.373	0.9893

Table S7. All calculated values pertaining to the Langmuir and Freundlich models for the simultaneous removal of PS-MPs and perfluorooctanoic acid (PFOA) by CNN4.

Gel	Langmuir			Freundlich		
	K_l (L/mg)	Q_{max} (mg/g)	R^2	K_f (mg/g)(L/mg)$^{1/n}$	n	R^2
$CNN4\_Q_{75}B_{300}$ **MP in the Mixture**	0.1112	478.4	0.9120	80.45	2.886	0.8899
$CNN4\_Q_{75}B_{300}$ **PFOA in the Mixture**	0.1048	134.6	0.8215	31.08	4.132	0.6543
$CNN4\_Q_{75}B_{300}$ **PFOA**	0.01195	146.8	0.9361	11.22	2.476	0.9518

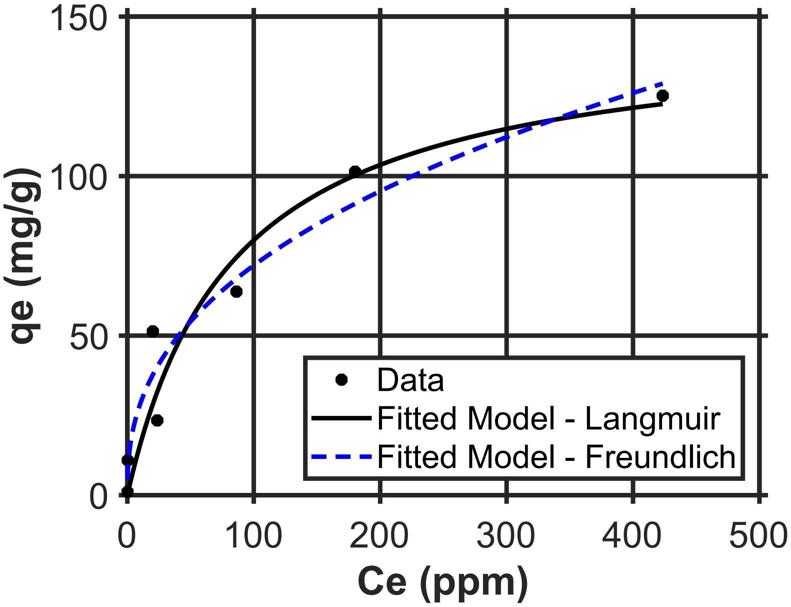

Figure S16. Adsorption isotherm and fitted model for perfluorooctanoic acid (PFOA) adsorption by CNN4.

5. Adsorption Mechanism Investigations

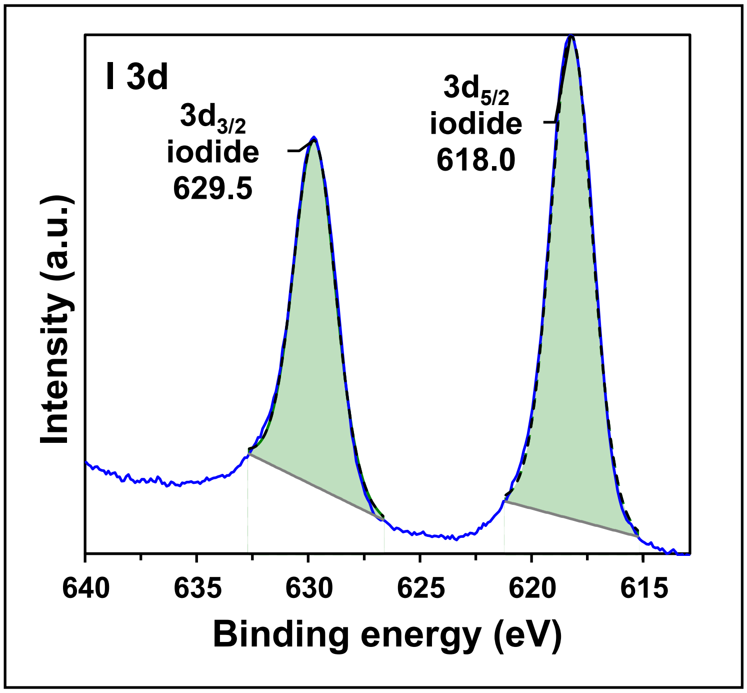

Figure S17. High resolution XPS spectrum of I 3d in CNN4 gel

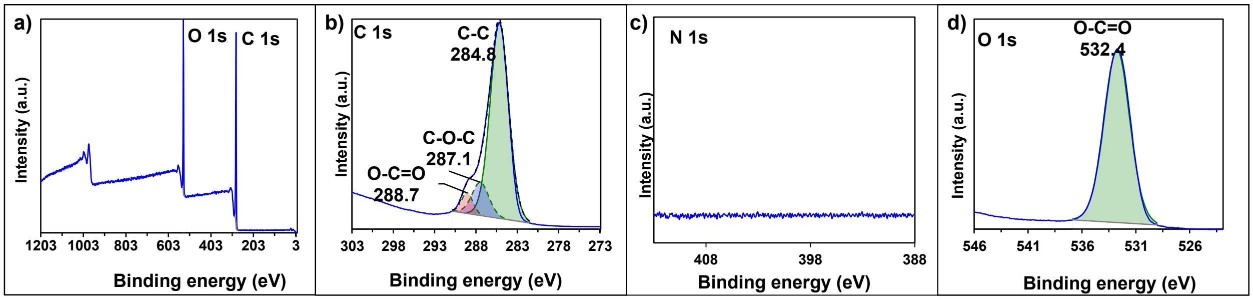

Figure S18. a) XPS general survey NN5. b) high resolution C1s. c) high resolution N1s. d) high resolution O1s

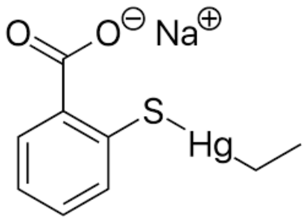

Figure S19. Chemical structure of thimerosal, used as a preservative for MP at a concentration of 0.02%.

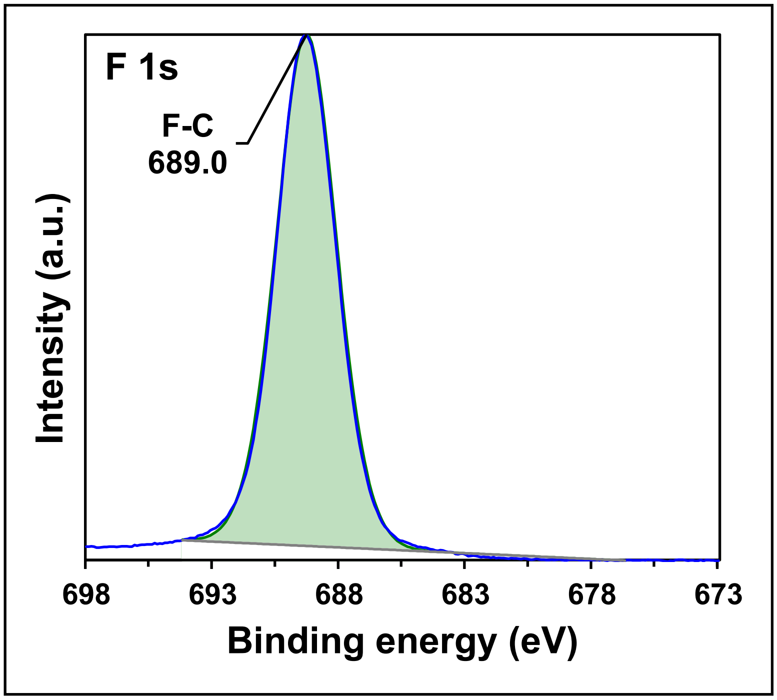

Figure S20. High resolution XPS spectrum of **F 1s** in CNN4 gel after adsorption of MP and PFOA

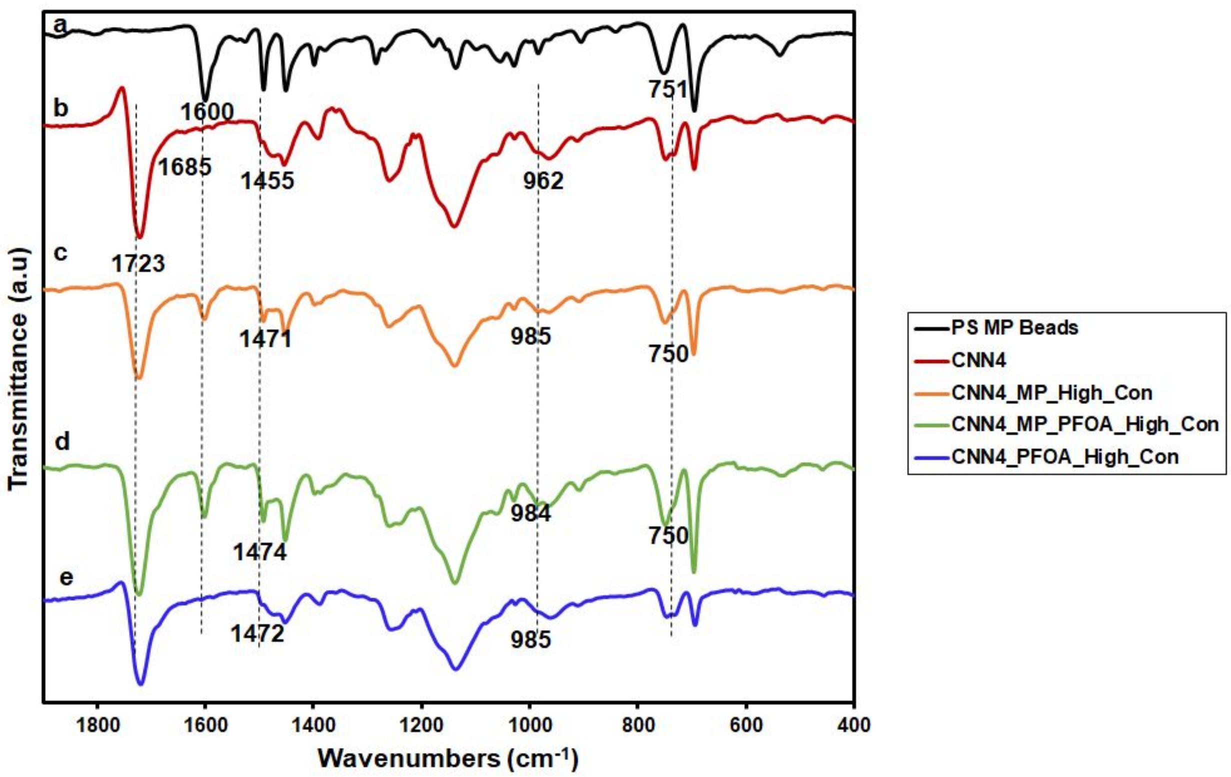

Figure S21. FTIR-ATR spectrum of CNN4, MP and PFOA before and after adsorption (wave number: 100-1900 cm-1)

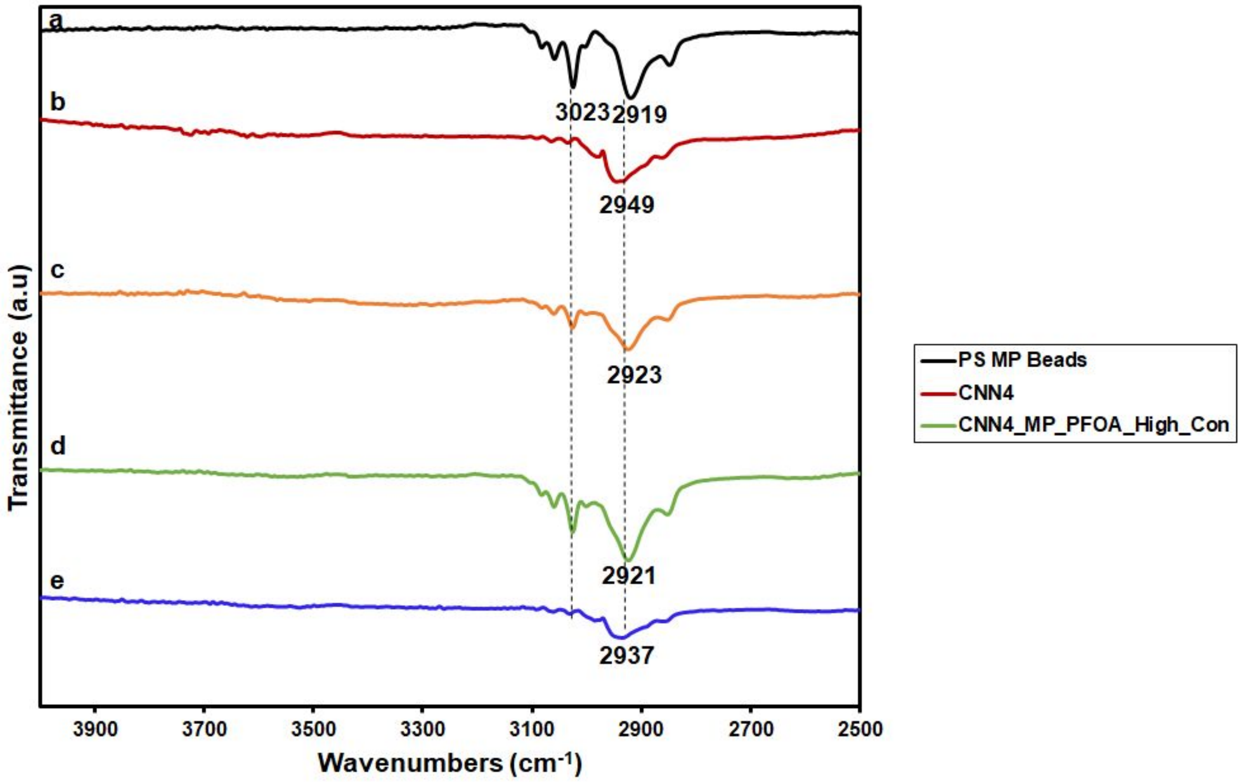

Figure S22. FTIR-ATR spectrum of CNN4, MP and PFOA before and after adsorption (wave number: 2500-4000 cm-1)

6. Reusability of CNN adsorbent

Initially, the gel was weighed to 30 mg after removing 100 ppm (MP), and placed in a 50 mL Round Bottom Flask (RBF). A 30 mL NaCl solution (1 M) was prepared and added to the flask. The mixture was stirred vigorously for 15 minutes using a high vortex setting.

Following the mixing, the solution was filtered through a Fritted Glass C Filter with a pore size of 30-50 micrometers, and the supernatant was collected for fluorescence measurement. This washing process was repeated eight times for the same gel sample and followed by eight times washing with DI water, a methanol rinse to aid drying. In addition various concentration of NaCl ranging from 100 mM to 6M and , 100 mM ammonium acetate, were tested for their effectiveness in MP desorption. Although higher NaCl salt concentrations improved desorption efficiency, complete removal was not achieved due to the strong charge interactions between the gel and MP.

This process was repeated for a total of three cycles, with only 8.86% of the gel's original adsorption capacity being lost. The original CNN4 removed 94.32% of 100 ppm of MP, while the results for subsequent cycles were as follows: second run: 86.75%, third run: 82.86%, and fourth run: 85.78%. The slight reduction in capacity is likely due to losses during filtration and the irreversible nature of certain gel-MP interactions. Despite this, CNN4 consistently demonstrated a high adsorption capacity, with no saturation point reached even when treating 100 ppm MP, emphasizing the gel’s potential for sustainable reuse.

Figures. S23 illustrating the gel removal efficiency cycles and the picture of before and after regeneration, under both room light and UV light, showed color changes, due to the desorption of MP and no physical changes on the gel supporting the gel’s durability across multiple cycles.

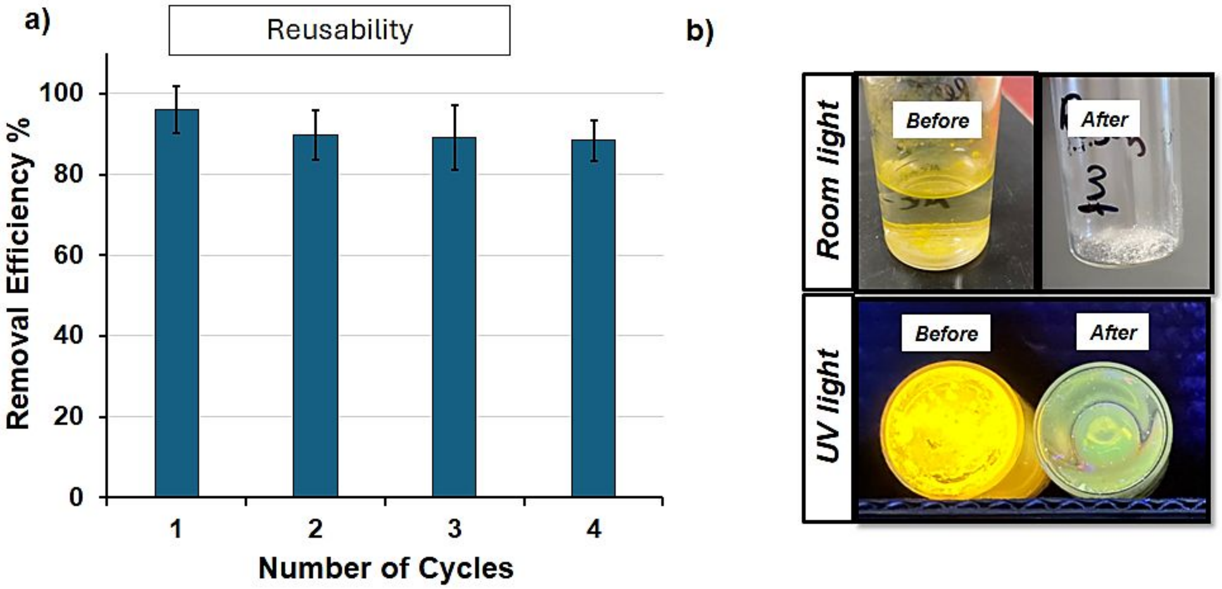

Figure S23. a) Reusability performance of CNN4 across multiple cycles. b) Visual comparison of the gel before and after the third regeneration cycle, shown under both room light and UV light.

7. Comparative analysis of microplastic removal techniques

Numerous comprehensive studies have been conducted to compare and report on the performance of various microplastic removal techniques, detailing metrics such as removal efficiency, adsorption capacity, and other key parameters. For instance, Liu et al. (2024)[5] published a thorough review that compiled and analyzed a wide range of techniques, providing detailed evaluations. Building on their findings, we have selected the best-performing methods from each category and integrated the most recent advancements to enhance the scope of this study. Additionally, Tipplook et al. (2024)[6] conducted an extensive study on PFAS removal, which has been referenced in our work to provide context and support for the comparative analysis. This study goes further by comparing these established techniques with the performance of our newly developed material, offering a comprehensive evaluation of its efficacy relative to existing methods.

Table S8.Comparative analysis of microplastic removal techniques

Material	Microplastic (Size µm)	Removal efficiency%	Reusability (#Runs)	Qe (mg/g)
**This work**				
CNNs	PS	96.6%	85.7(4th)	1865
**Carbon-based[5]**				
graphite carbon-carbon nanofiber aerogel	PS (0.147-0.400)	93.2 -98.5 %	-	-
graphene-like carbon assembled layered double oxide material	PS (0.080)	75-100%	90% (5th)	209.39
**Chitosan-based**				
Chitosan nanofiber sponge	PET (48.7)	80.1%	-	-
chitin-based sponge modified with positively charged chitosan	PS<br>PS-COOH<br>PS-NH_2<br>(1)	92.1%<br>81.3%<br>91.3%	88.9%(2nd)<br>-<br>88.9%(2nd)	8.28<br>6.75<br>-
**Cellulose-based**				
modified CNF/PVA/EPTMAC aerogel	PS	~98%	83.1%(2ND)	146.38

directional CNF/3-glycidyloxypropyltrimethoxysilane/PEI aerogel	PS	~100%	85%(2ND)	117.04
**Protein-based**				
Oat protein-based sponge	PS	75%	~60% (4th)	5.7
Starch-gelatin sponge	PMMA	90%	-	20.43
**MOF-based**				
2D MOF@C@FeO	PS	~100%	90%(6th)	725.5
Cr-MOF/MIL-101	PS	96%	81% (2nd)	800
**Magnetic-based**				
Fe3O4/halloysite-PDMS/sponge	PP	99.98%	99% (50th)	24.3
Magnetic PDMS-Ni foam	PS	94.87%	97.7% (10th)	-
**Coagulation /Flocculation**[7]				
Coagulants aluminum chlorohydrate (PAC) and polyacrylamide (PAM)	PS (50-1000 nm)	98%	-	-
**Microrobots**				
Ag@Bi2WO6/Fe3O4 Microrobots (Low energy VIS)[8]	PS, PMMA, SiO2 (2-10 μm)	98%	-	-

Magnetically driven liquid metal microrobot[9]	Microplastics (2 μm), nanoplastics (30 nm)	80%	-	-

8. References

(1) Shahrokhinia, A.; Rijal, S.; Sonmez Baghirzade, B.; Scanga, R. A.; Biswas, P.; Tafazoli, S.; Apul, O. G.; Reuther, J. F. Chain extensions in PhotoATRP-induced self-assembly (photoATR-PISA): a route to ultrahigh solids concentrations and click nanoparticle networks as adsorbents for water treatment. *Macromolecules* **2022,** *55* (9), 3699-3710.
(2) Hwang, N.; Barron, A. R. BET surface area analysis of nanoparticles. *The connexions project* **2011**, 1-11.
(3) Budd, P. M.; Makhseed, S. M.; Ghanem, B. S.; Msayib, K. J.; Tattershall, C. E.; McKeown, N. B. Microporous polymeric materials. *materials today* **2004,** *7* (4), 40-46.
(4) Misra, A.; Zambrzycki, C.; Kloker, G.; Kotyrba, A.; Anjass, M. H.; Franco Castillo, I.; Mitchell, S. G.; Güttel, R.; Streb, C. Water purification and microplastics removal using magnetic polyoxometalate-supported ionic liquid phases (magPOM-SILPs). *Angewandte Chemie International Edition* **2020,** *59* (4), 1601-1605.
(5) Liu, Q.; Khor, S. M. Emerging absorption-based techniques for removing microplastics and nanoplastics from actual water bodies. *TrAC Trends in Analytical Chemistry* **2024,** *170*, 117465.
(6) Tipplook, M.; Hisama, K.; Koyama, M.; Fujisawa, K.; Hayashi, F.; Sudare, T.; Teshima, K. Cation-Doped Nanocarbons for Enhanced Perfluoroalkyl Substance Removal: Exotic Bottom-Up Solution Plasma Synthesis and Characterization. *ACS Applied Materials & Interfaces* **2024,** *16* (45), 61832-61845.
(7) Zhang, Y.; Wang, X.; Li, Y.; Wang, H.; Shi, Y.; Li, Y.; Zhang, Y. Improving nanoplastic removal by coagulation: Impact mechanism of particle size and water chemical conditions. *Journal of Hazardous Materials* **2022,** *425*, 127962.
(8) Wang, Z.; Xu, L.; Cai, X.; Yu, T. Low-Energy Photoresponsive Magnetic-Assisted Cleaning Microrobots for Removal of Microplastics in Water Environments. *ACS Applied Materials & Interfaces* **2024,** *16* (45), 61899-61909.
(9) Wu, X.; Peng, X.; Ren, L.; Guan, J.; Pumera, M. Reconfigurable Magnetic Liquid Metal Microrobots: A Regenerable Solution for the Capture and Removal of Micro/Nanoplastics. *Advanced Functional Materials* **2024,** *34* (51), 2410167.
